# Supplementary figures and images for: Potential impacts of climate change on geographical distribution of three primary vectors of African Trypanosomiasis in Tanzania’s Maasai Steppe: G. m. morsitans, G. pallidipes and G. swynnertoni
Source: PLoS Negl Trop Dis. 2021 Feb 11;15(2):e0009081. doi: 10.1371/journal.pntd.0009081 (PMC7904224; doi:10.1371/journal.pntd.0009081)

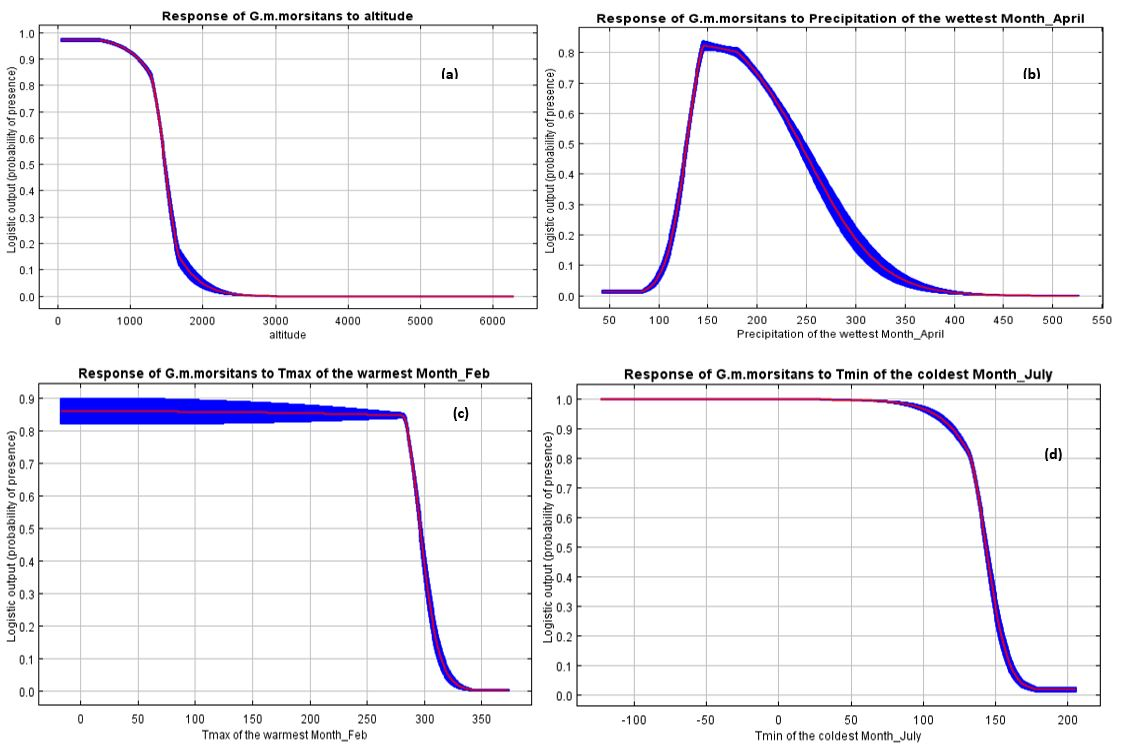

Supplement: S1 Fig — Temperature is reported in 0C * 10. (TIF) [file pntd.0009081.s001.tif]

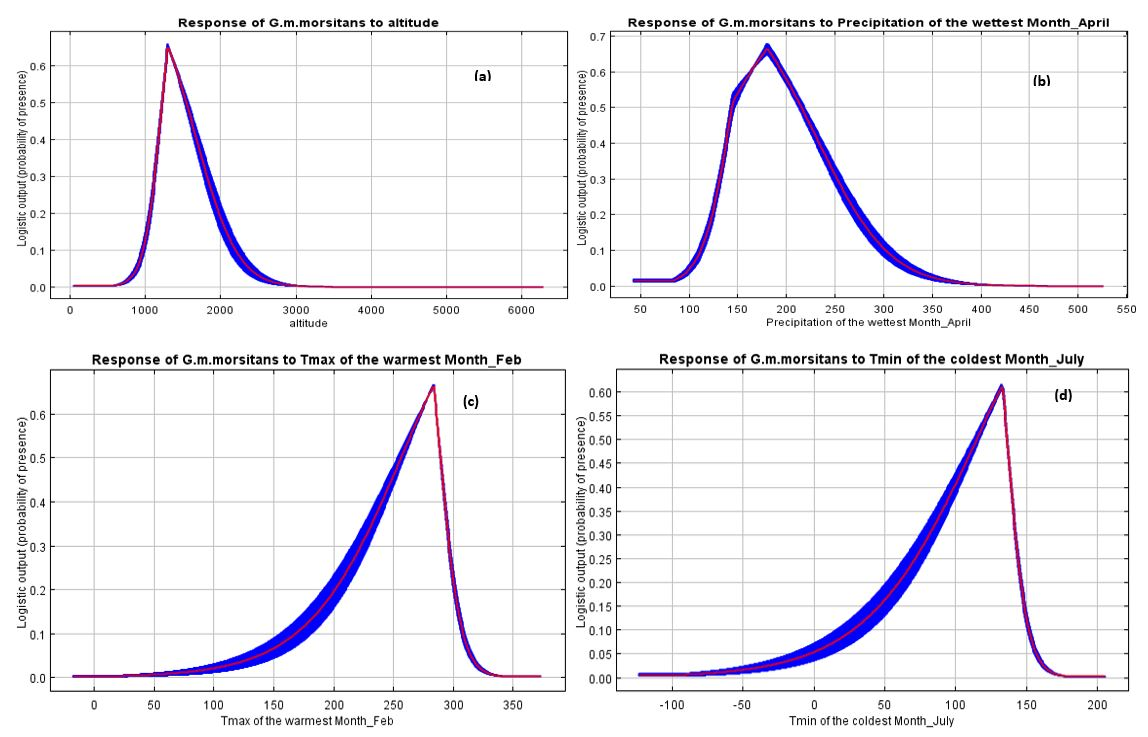

Supplement: S2 Fig — Temperature is reported in 0C * 10. (TIF) [file pntd.0009081.s002.tif]

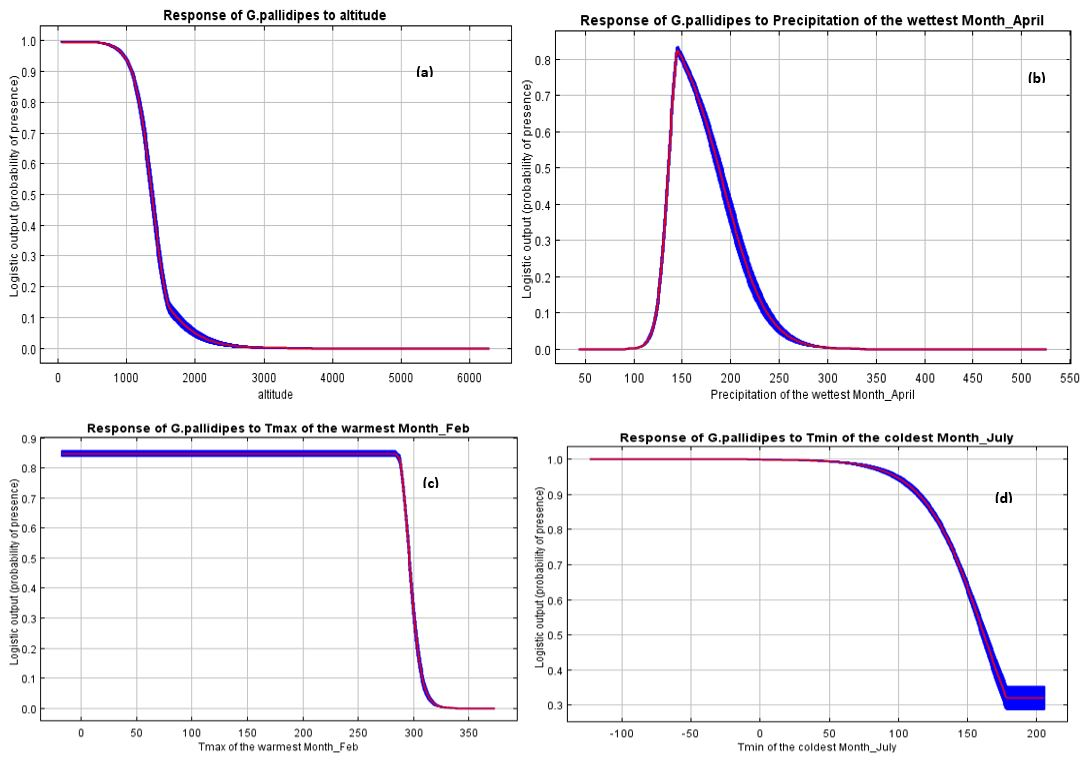

Supplement: S3 Fig — Variable performance is assessed via the variables’ impact to training and test gain (top and middle) and AUC (bottom). (TIF) [file pntd.0009081.s003.tif]

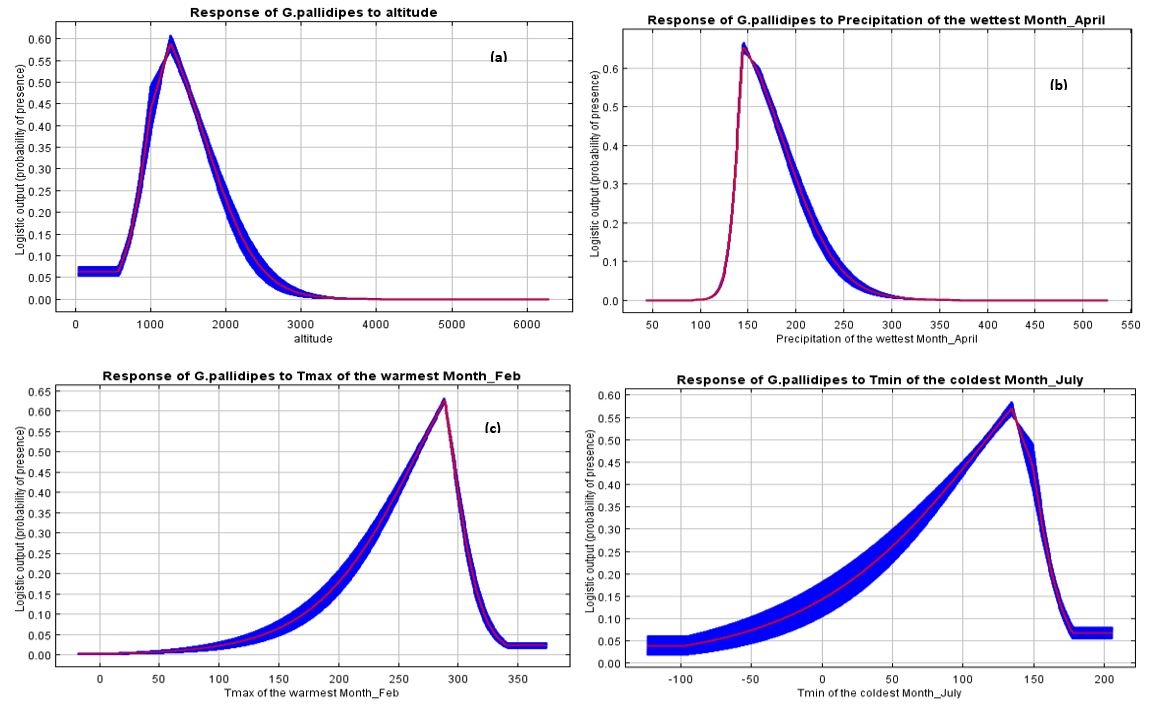

Supplement: S4 Fig — Temperature is reported in 0C * 10. (TIF) [file pntd.0009081.s004.tif]

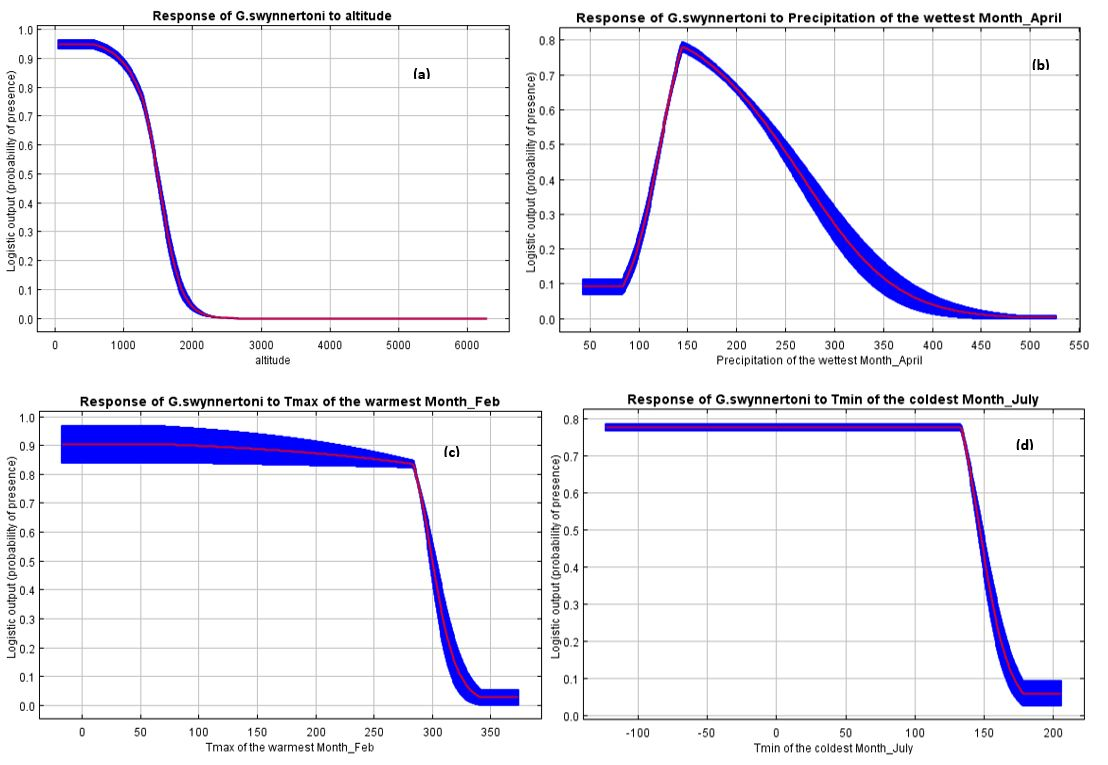

Supplement: S5 Fig — Temperature is reported in 0C * 10. (TIF) [file pntd.0009081.s005.tif]

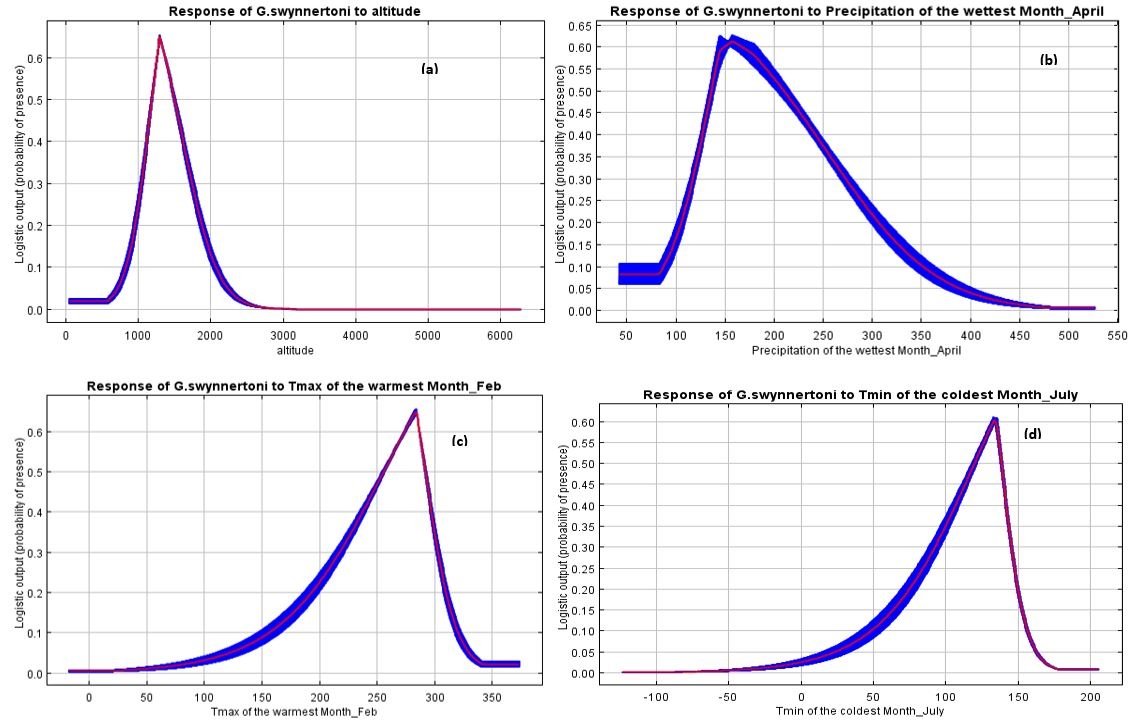

Supplement: S6 Fig — Variable performance is assessed with training and test gain (top and middle) and AUC (bottom). (TIF) [file pntd.0009081.s006.tif]

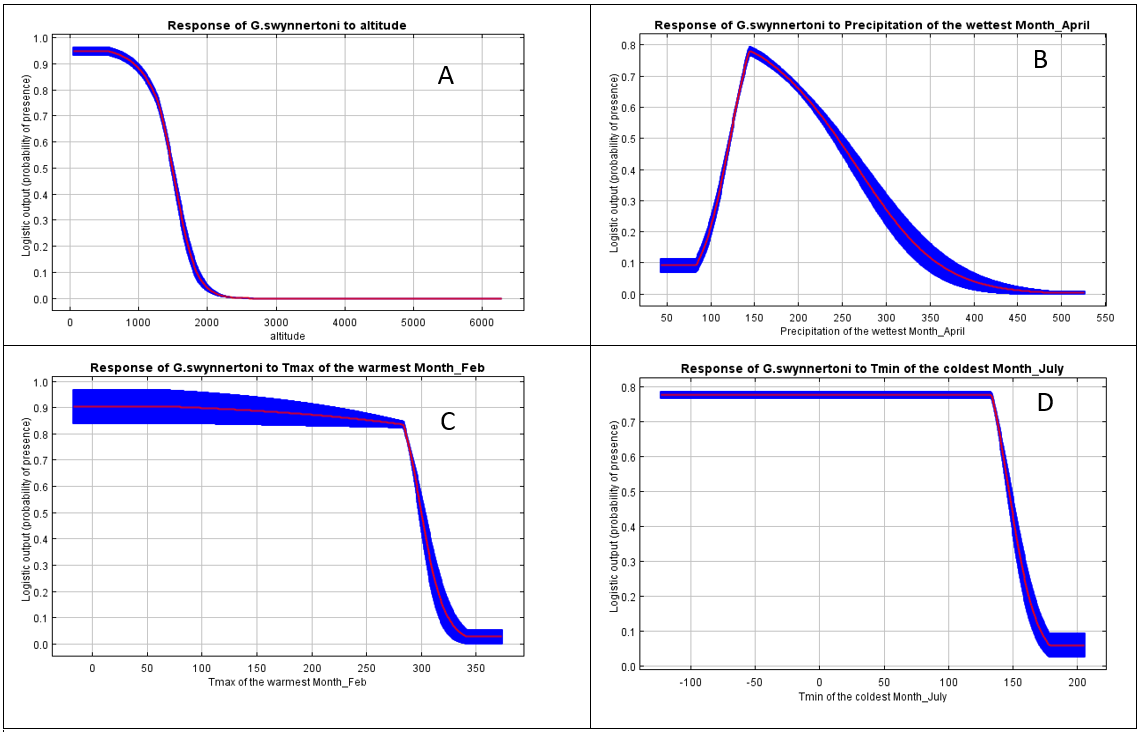

Supplement: S7 Fig — Temperature is reported in 0C * 10. (TIF) [file pntd.0009081.s007.tif]

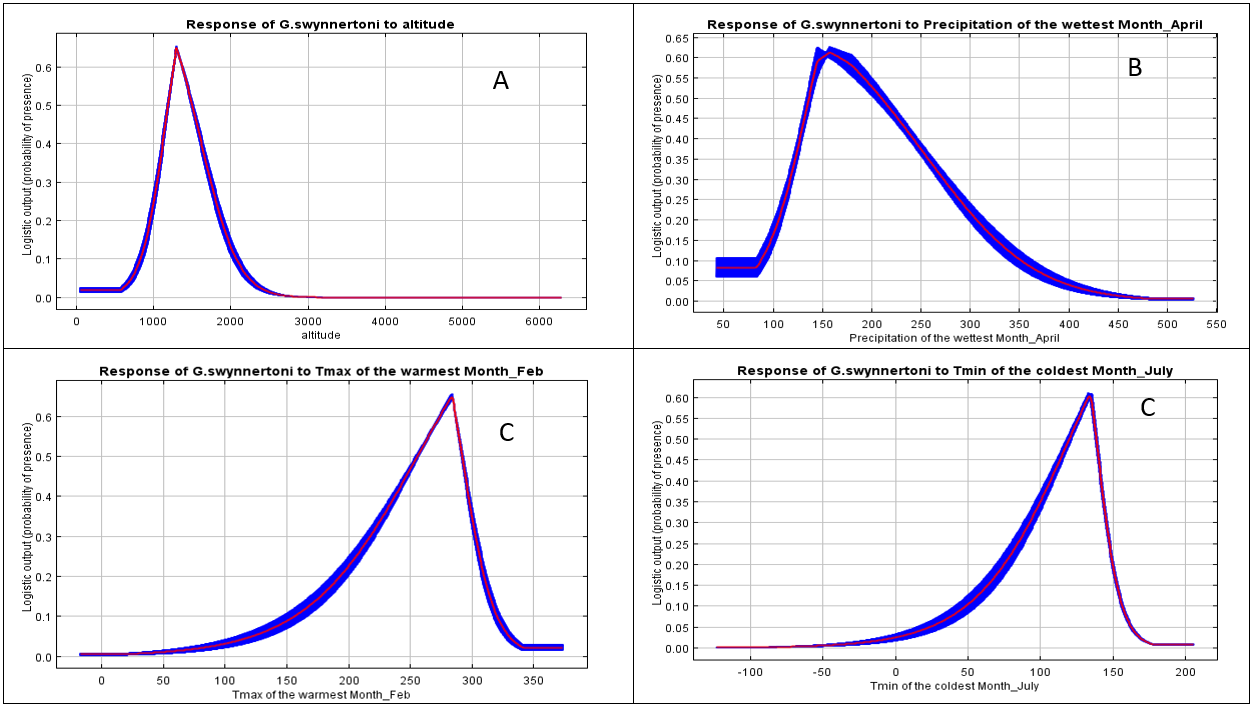

Supplement: S8 Fig — Temperature is reported in 0C * 10. (TIF) [file pntd.0009081.s008.tif]

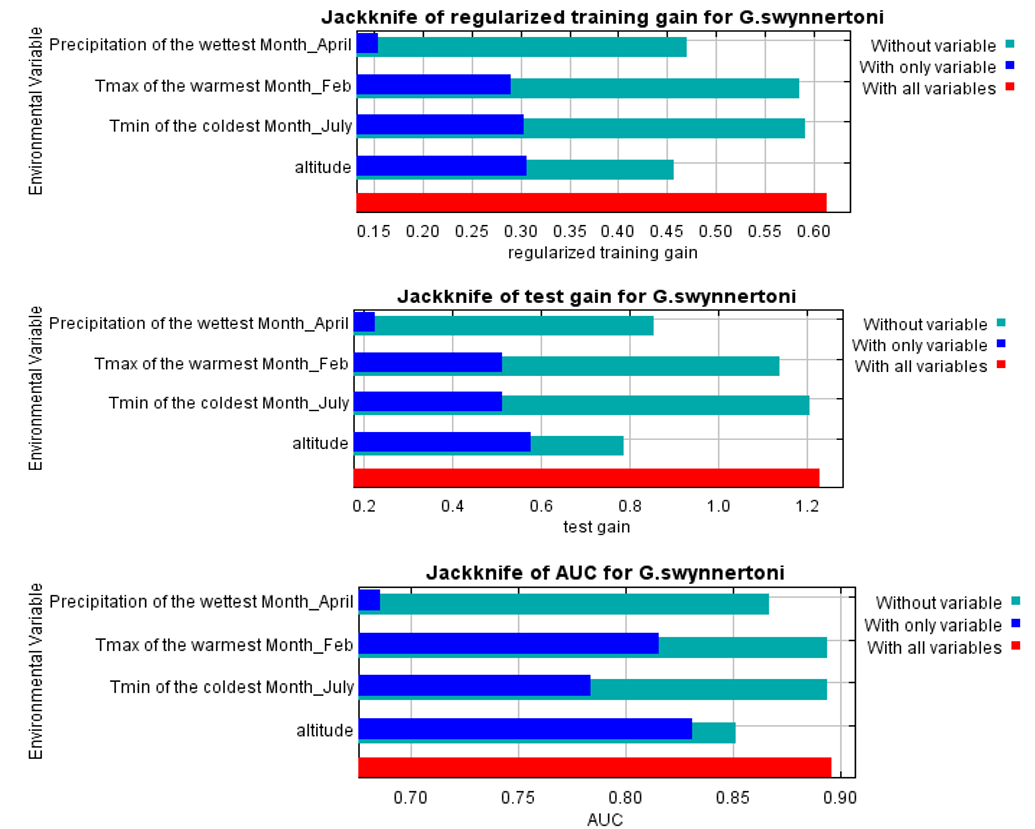

Supplement: S9 Fig — Variable performance is assessed with training and test gain (top and middle) and AUC (bottom) (TIF) [file pntd.0009081.s009.tif]
