# Supplementary material for: Potential impacts of climate change on geographical distribution of three primary vectors of African Trypanosomiasis in Tanzania’s Maasai Steppe: G. m. morsitans, G. pallidipes and G. swynnertoni
Source: PLoS Negl Trop Dis. 2021 Feb 11;15(2):e0009081. doi: 10.1371/journal.pntd.0009081 (PMC7904224; doi:10.1371/journal.pntd.0009081)
Supplement: Attachment — Submitted filename: Comments on PNTD.pdf [file pntd.0009081.s010.pdf]

Comments on PNTD-D-20-00736

The study outlined in the manuscript deployed MaxEnt species distribution modelling (SDM) and ecological niche modelling tools to depict likely distribution patterns of three *G. m. morsitans, G. pallidipes* and *G. swynnertoni* tsetse fly species in Tanzania Maasai Steppe based on the current climate profile and predicted change by mid-century under Representative Concentration Pathway (RCP) 4.5 scenario. The results predict that habitable areas may decrease by up to 23.13%, 12.9% and 22.8% for the three species, respectively, by 2050.

The study appears to have been well conducted. However, it requires significant editorial correction or improvement, and specially the Discussion part, which appears to be too wordy and thematically repetitive.

Line 66: 'dairy', not 'diary'.

Line 107: 'practising' instead of 'practicing'?

Lines 127-30, 2nd part of the sentence: change the second part of the sentence from 'including to predict the probability of occurrence of species across space and time..'.

Line 140: change 'plays' to 'play'.

Line 141: '…..since blood meals are the only tsetse fly nutrition…….'.

Line 209-213: '…… the model showed that currently 32% (19,225 km^2) of the entire Maasai Steppe (≈ 60,000 km^2) has suitable climate for *G. m. morsitans*, but this would shrink to 7.4% (4,447.34 km^2) by 2050'.

Line 258-262: The three sentences appear to repeat more or less the same message……it can be condensed into one or two sentences.
