# Supplementary material for: Potential impacts of climate change on geographical distribution of three primary vectors of African Trypanosomiasis in Tanzania’s Maasai Steppe: G. m. morsitans, G. pallidipes and G. swynnertoni
Source: PLoS Negl Trop Dis. 2021 Feb 11;15(2):e0009081. doi: 10.1371/journal.pntd.0009081 (PMC7904224; doi:10.1371/journal.pntd.0009081)
Supplement: Attachment — Submitted filename: PNTD-D-20-00736_Agapitus.pdf [file pntd.0009081.s011.pdf]

PLOS Neglected Tropical Diseases

Potential Impacts of Climate Change on Geographical Distribution of Three Primary Vectors of African Trypanosomiasis in Tanzania Maasai Steppe; G. m. morsitans, G. pallidipes and G. swynnertoni

--Manuscript Draft--

**Manuscript Number:**	PNTD-D-20-00736
**Full Title:**	Potential Impacts of Climate Change on Geographical Distribution of Three Primary Vectors of African Trypanosomiasis in Tanzania Maasai Steppe; G. m. morsitans, G. pallidipes and G. swynnertoni
**Short Title:**	Climate Change and Vectors of African Trypanosomiasis
**Article Type:**	Research Article
**Keywords:**	Climate change; Maasai Steppe; tsetse fly; African Trypanosomiasis; MaxEnt; climate envelope; SDM

Abstract:

In Maasai Steppe, public health and economy are threatened by African Trypanosomiasis; a debilitating and fatal disease to livestock (African Animal Trypanosomiasis -AAT) and humans (Human African Trypanosomiasis - HAT), if not treated. Tsetse fly is a primary vector for both HAT and AAT and climate is an important predictor of their occurrence and parasites they carry. While understanding tsetse fly distribution is essential for informing vector and disease control strategies, existing distribution maps are old and were based on coarse spatial resolution data which [illegible]t useful in understanding vector and disease dynamics necessary to design and implement fit for purpose mitigation strategies. Also, assertion that climate change is altering tsetse fly distribution in Tanzania lacks empirical evidence. Despite tsetse fly posing public health risks and economic hardship, no study has modelled their distributions at a scale needed for local planning. This study used MaxEnt species distribution modelling (SDM) and ecological niche modeling tools to predict potential distribution of three tsetse fly species in Tanzania Maasai Steppe from current climate information and project their distributions to midcentury climatic conditions under representative concentration pathways (RCP) 4.5 scenarios. Current climate results predicted that G. m. morsitans, G. pallidipes and G swynnertoni cover 19,225 km^2, 7,113 km^2 and 32,335 km^2 and future prediction indicated that by the year 2050, the habitable area may decrease by up to 23.13%, 12.9% and 22.8% of current habitable area respectively. This information can serve as a useful predictor of potential HAT and AAT hotspots and inform surveillance strategies. Distribution maps generated by this study can be useful in guiding tsetse fly control managers, health, livestock and wildlife officers when setting surveys and surveillance programs. The maps can also inform protected area managers of potential encroachment due to shrinkage of tsetse fly habitats in the protected area.

**Additional Information:**	
**Question**	**Response**
**Financial Disclosure**<br><br>Enter a financial disclosure statement that describes the sources of funding for the work included in this submission. Review the submission guidelines for detailed requirements. View published research articles from *PLOS NTDs* for specific examples.<br><br>This statement is required for submission and **will appear in the published article** if	The author(s) received no specific funding for this work.

the submission is accepted. Please make sure it is accurate.<br><br>**Unfunded studies**<br><br>Enter: *The author(s) received no specific funding for this work.*<br><br>**Funded studies**<br><br>Enter a statement with the following details:<br>• Initials of the authors who received each award<br>• Grant numbers awarded to each author<br>• The full name of each funder<br>• URL of each funder website<br>• Did the sponsors or funders play any role in the study design, data collection and analysis, decision to publish, or preparation of the manuscript?<br>• **NO** - Include this sentence at the end of your statement: *The funders had no role in study design, data collection and analysis, decision to publish, or preparation of the manuscript.*<br>• **YES** - Specify the role(s) played.<br><br>* typeset	
**Competing Interests**<br><br>Use the instructions below to enter a competing interest statement for this submission. On behalf of all authors, disclose any competing interests that could be perceived to bias this work—acknowledging all financial support and any other relevant financial or non-financial competing interests.<br><br>This statement **will appear in the published article** if the submission is accepted. Please make sure it is accurate. View published research articles	The authors have declared that no competing interests exist.

from *PLOS NTDs* for specific examples.<br><br>**NO authors have competing interests**<br><br>Enter: *The authors have declared that no competing interests exist.*<br><br>**Authors with competing interests**<br><br>Enter competing interest details beginning with this statement:<br><br>*I have read the journal's policy and the authors of this manuscript have the following competing interests: [insert competing interests here]*<br><br>* typeset	
**Data Availability**<br><br>Authors are required to make all data underlying the findings described fully available, without restriction, and from the time of publication. PLOS allows rare exceptions to address legal and ethical concerns. See the PLOS Data Policy and FAQ for detailed information.<br><br>A Data Availability Statement describing where the data can be found is required at submission. Your answers to this question constitute the Data Availability Statement and **will be published in the article**, if accepted.	Yes - all data are fully available without restriction

**Important:** Stating 'data available on request from the author' is not sufficient. If your data are only available upon request, select 'No' for the first question and explain your exceptional situation in the text box.<br><br>Do the authors confirm that all data underlying the findings described in their manuscript are fully available without restriction?	
**Describe where the data may be found in full sentences. If you are copying our sample text, replace any instances of XXX with the appropriate details.**<br><br>• If the data are **held or will be held in a public repository**, include URLs, accession numbers or DOIs. If this information will only be available after acceptance, indicate this by ticking the box below. For example: *All XXX files are available from the XXX database (accession number(s) XXX, XXX.*).<br>• If the data are all contained **within the manuscript and/or Supporting Information files**, enter the following: *All relevant data are within the manuscript and its Supporting Information files.*<br>• If neither of these applies but you are able to provide **details of access elsewhere**, with or without limitations, please do so. For example:<br><br>*Data cannot be shared publicly because of [XXX]. Data are available from the XXX Institutional Data Access / Ethics Committee (contact via XXX) for researchers who meet the criteria for access to confidential data.*<br><br>*The data underlying the results presented in the study are available*	At present data are available by writting at happyjackson.nnko@gmail.com

from (include the name of the third party and contact information or URL).<br>• This text is appropriate if the data are owned by a third party and authors do not have permission to share the data.<br><br>* typeset	
Additional data availability information:	Tick here if your circumstances are not covered by the questions above and you need the journal's help to make your data available.

Manuscript

Click here to download Manuscript Ptential distribution of Tsetse_Manuscript MAY 2020_01.docx

Potential Impacts of Climate Change on Geographical Distribution of Three Primary Vectors of African Trypanosomiasis in Tanzania Maasai Steppe; *G. m. morsitans, G. pallidipes and G. swynnertoni*

Happiness J. Nnko[1*], Paul S. Gwakisa[2¶], Anibariki Ngonyoka[1¶] Calvin Sindato [3&4,] & Anna Estes [5¶],

[1]University of Dodoma, Dodoma Tanzania,

[2]Sokoine University of Agriculture, Morogoro, Tanzania

[3]National Institute for Medical Research, Tabora, Tanzania,

[4]Southern African Centre for Infectious Disease Surveillance, Morogoro Tanzania &.

[5]Carleton University, Canada.

*Corresponding author

E-mail:happyjackson.nnko@gmail.com

Abstract

In Maasai Steppe, public health and economy are threatened by African Trypanosomiasis; a debilitating and fatal disease to livestock (African Animal Trypanosomiasis -AAT) and humans (Human African Trypanosomiasis - HAT), if not treated. Tsetse fly is a primary vector for both HAT and AAT and climate is an important predictor of their occurrence and the parasites they carry. While understanding tsetse fly distribution is essential for informing vector and disease control strategies, existing distribution maps are old and were based on coarse spatial resolution data which is not useful in understanding vector and disease dynamics necessary to design and implement fit

for purpose mitigation strategies. Also, assertion that climate change is altering tsetse fly distribution in Tanzania lacks empirical evidence. Despite tsetse fly posing public health risks and economic hardship, no study has modelled their distributions at a scale needed for local planning. This study used MaxEnt species distribution modelling (SDM) and ecological niche modeling tools to predict potential distribution of three tsetse fly species in Tanzania Maasai Steppe from current climate information and project their distributions to midcentury climatic conditions under representative concentration pathways (RCP) 4.5 scenarios. Current climate results predicted that *G. m. morsitans, G. pallidipes* and *G swynnertoni* cover 19,225 km^2, 7,113 km^2 and 32,335 km^2 and future prediction indicated that by the year 2050, the habitable area may decrease by up to 23.13%, 12.9% and 22.8% of current habitable area respectively. This information can serve as a useful predictor of potential HAT and AAT hotspots and inform surveillance strategies. Distribution maps generated by this study can be useful in guiding tsetse fly control managers, health, livestock and wildlife officers when setting surveys and surveillance programs. The maps can also inform protected area managers potential encroachment due to shrinkage of tsetse fly habitats in the protected area.

Key Words: *Climate change, Maasai Steppe, tsetse fly, MaxEnt, SDM,*

Authors' summary

Spatial variation of African Trypanosomiasis burden depends on distribution of biotopes necessary for tsetse flies to thrive. Therefore, mapping the occurrence of the tsetse fly species is a useful predictor of African Trypanosomiasis transmission risk areas. Climate is a major determining factor for occurrence and survival of tsetse fly, the vector responsible for both HAT and AAT. Since resources for prevention and control of tsetse fly species and the disease they transmit are generally scarce in endemic settings, understanding potential impacts of climate

change on tsetse fly species distribution in space and time is essential for informing coherent strategies for vector and disease control at a local scale.

Introduction

Most climate change predictions show an upward trend in temperature for at least the next nine decades [1], but there is uncertainty with different climate models predicting different magnitudes of warming. On average, global temperature is expected to rise by 0.8-2.6^{0}C and by 1.5-3^{0}C in Africa by the year 2050 [2]. Such increases have potential to cause species habitat modification including range expansion or contraction in addition to altering their relationships with bio-physical environment. The influence of climate change on species distribution is supported by evidence from fossil records [3] and observed trends from the twentieth to twenty first centuries on species range shifts. For example, it is estimated that a change in 1^{0}C will lead to range shifts of 160km of ecological zone on earth, implying that if the globe will warm by 3^{0}C by the year 2100, the flora and fauna of the North Pole will move approximately 480 km northward to remain within their thermal tolerances [4-5]. Some species of butterflies in Europe have been reported to shift further north as those zones become more habitable [6-8]. Predicted rise in temperature is also expected to transform dynamics of vector-borne diseases including African Trypanosomiasis, either, by altering the vectors and pathogens geographical range, or their development and mortality rates [9-12].

Tsetse fly occurs in Sub-Saharan Africa and their distribution is influenced by climate, vegetation and hosts. Climate, particularly temperature is considered a major driver as it influences all others factors that determine tsetse occurrence. Trypanosomiasis remains a debilitating and fatal disease to livestock and humans, if left untreated. For instance, trypanosomiasis in livestock

causes loss of over 4 billion USD due to 70% reduction of cattle density, 50% reduction in diary and meat sales, 20% reduction in calving rates, and 20% increases in calf mortality in Sub-Saharan Africa [13]. In Tanzania, tsetse fly occurs in over 65% of rangeland savannah ecosystems [14], exposing about 4 million people in rural communities to the risk of sleeping sickness and causing loss of approximately eight million USD annually due to nagana induced low livestock productivity [15-17]. Since dynamics of African trypanosomiasis is a function of tsetse fly competence, and the ecology and behavior of available hosts, spatial variation of disease burden depending on the distribution of biotopes necessary for tsetse flies to thrive is expected.

Trends in climate change and associated socioeconomic transformation is anticipated to continue altering tsetse fly habitats in Tanzania rangelands. Nonetheless, empirical evidence to support the assertion about change in tsetse fly species distribution as a result of climate change is lacking in the country. Also, information that could aid tsetse control planning for future preparedness is rare to find in the country and absent at local scales. In the Maasai Steppe, for instance, knowledge on tsetse fly spatial variation is often based on old and course data and not publicly available.

Various scientific approaches have been used to understand the potential impacts of climate on spatial and temporal distribution of disease vectors. Some of the approaches include climate envelope models and correlations between climatic variables and vectors [18-21]. Climate envelopes are species distribution models that use climate data to define climate suitability for species to occur [22]. Specifically, these models rely~~ies~~ on statistical correlations between species distributions points and their associated climate parameters to define a species' envelope of tolerance around existing ranges thereby delineating a ‘climate envelope’ within which species

thrive [19;22]. Compared to mechanistic models, climate envelope models do not incorporate data other than occurrence and environmental related data; so they do not predict fitness variation across climate gradients [23].

There have been research that studied risk of African Trypanosomiasis and tsetse fly burden in the Maasai Steppe [24-31]. However, none of these established potential impacts of climate change on distribution of tsetse. To fill this gap, a general question on what is the potential impact of climate change on the distribution of common *Glossina* species found in the study area was investigated. This study adopted a general definition of climate envelopes in which models were built using climate variables to define areas that have suitable climate for the tsetse fly and model their distribution based on current climate under which they have been observed. Prediction for future distribution was carried out to understand how African Trypanosomiasis transmission hotspots might change under future climate scenarios. This information may help stakeholders to allocate scarce resources in preventing African Trypanosomiasis by implementing more targeted interventions. This study also may form a basis for a large national and regional scale prediction of future African Trypanosomiasis transmission hotspots.

Methodology

Study area

This study was carried out in the Tanzania Maasai Steppe, located between 1.5 to 5° South latitude and 35 to 37° East Longitude (Fig 1). It covers an area of more than 60,000 km^2 with a population of over 600,000 people, mainly practicing pastoralism and to a lesser extent, agro-pastoralism. The region is semi-arid and a human-wildlife-livestock system, receiving up to 500 mm of rainfall per annum. Rainfall patterns dictate movement of pastoralists and their herds and

wildlife in search for water and pastures. These movements increases the likelihood of disease transmission between domestic animals, people and wildlife [3].

Data Collection

Species occurrence and background data

This study targeted three *Glossina* species *G.m.morsitans*, *G.pallidipes* and *G. swynnertoni* commonly found in the Maasai Steppe [27, 28, 29]. Abundance data were collected through entomological field surveys carried out once in the dry season, November 2015 and once in the wet season, May 2016. A total of 99 baited epsilon traps [33] were placed in Simanjiro and Monduli districts. Traps were deployed in stratified random subsampling of the major vegetation types [34] at a distance of at least 200m apart [16, 33]. At each trapping site, numbers of tsetse flies caught and geographical coordinates were recorded using hand-held Global Positioning System (GPS). The collected abundance data was converted to presence data for each of the GPS locations, yielding a total of 32, 59 and 29 unique occurrence points for *G.m.morsitans*, *G. pallidipes* and *G. swynnertoni*, respectively, after eliminating duplicate records resulted from multiple entries for a particular season. Duplicate records were removed using ecological niche modelling tools (ENMTools) software version 1.4.3 [35]. The occurrence data were used with climate predictor variables as input in MaxEnt (v 3.3.3k) [36], to create climate envelope models for the three species. MaxEnt is a species distribution model developed to work with presence-only data, and has been widely used in modelling and mapping species distributions [37], including to predict the probability of occurrence of species across space and time in areas that have not been sampled [36, 38]. Since dispersal of tsetse flies is dependent on availability of suitable hosts, and the study area is home to numerous hosts (wildlife and livestock), the study assumed that all

districts of Maasai Steppe were potential for attracting tsetse flies. For this reason, background data were sampled from the whole study area [38-40].

Climate layers

Predictive models for tsetse fly species distribution were made using the occurrence data and current climate variables (Table 1). The initial candidate layers considered in the model were elevation, precipitation of the wettest month (April), mean maximum temperature of the warmest month (February), mean maximum temperature of the driest month (September) and mean minimum temperature of the coldest month (July). Both maximum and minimum temperature affects tsetse fly activity patterns and plays an important role in determining the development of tsetse flies and trypanosomes at each life stage [41-42]. Since blood meals is the only known tsetse fly nutrition, no information is known on effects of precipitation on tsetse fly species except reports that indicate fluctuation of abundance during rainy season [25, 27, 43, 44]. However, it is thought that rainfall, apart from maintaining vegetation and humidity for tsetse fly to thrive, it also affects tsetse fly species indirectly by causing local flooding which may drown pupae that are buried in loose soil [45] and so it was included in predictor variables. Elevation, which is a proxy for temperature, was also used as a predictor variable in order to gain insight regarding the potential altitude limit for tsetse fly species to thrive. Although land cover/use, density of animals also influence tsetse fly distribution in space and time [16], this information was not included in the study due to inconsistency of available data. Models created using current climate variables were mapped on to future climate layers to understand how changing climate might influence tsetse distribution and thereby African Trypanosomiasis transmission risk. For the future climate projection scenario (year 2050), this study used 833.33m resolution Coupled Model Inter-comparison Project (CMIP5) global circulation model (GCM).

Table 1: Candidate covariates tested used in initial model runs, and the bolded ones used in the best-performing MaxEnt models

Variable	Type	Units	Resolution	source
**Precipitation of the wettest month (April)**	**Continuous**	**ml**	**833.33m**	**http://www.worldclim.org**
**Mean maximum temperature of the warmest month (April)**	**Continuous**	**^{0}C*10**	**833.33m**	**http://www.worldclim.org**
**Mean minimum temperature of the coolest month (July)**	**Continuous**	**^{0}C*10**	**833.33m**	**http://www.worldclim.org**
**Altitude/elevation**	**Continuous**	**msl**	**833.33m**	**http://www.worldclim.org**
Mean maximum temperature of the driest month (September)	Continuous	**^{0}C*10**	833.33m	http://www.worldclim.org

Of the many possible GCMs to use, CMIP5 was chosen because the CMIP5models are relatively more advanced (fine-tuned) and they use RCP scenario compared to previous GCMs that were released in or before 2010. In particular, the climate system model from Beijing Climate Center (BCC-CSM1-1) was used and the RCP 4.5 was selected for this study. The BCC-CSM1 was chosen for this analysis because it~~’s~~ is among the models that have been suggested to capture the key processes relevant to our study area [46]. Although there is uncertainty associated with any future climate scenario, these data provide reasonable predictions that can be useful for planning.

Modelling procedures

In order to minimize the use of correlated variables that may mask contribution of individual variables and cause difficulties in results interpretation [37, 47], pairwise collinearity tests of predictor variables was performed using ENMTool 1.4.3[35-36]. Temperature variables and altitude were highly correlated but mean minimum and maximum temperature of coldest and

warmest month respectively were maintained because of their high biological relevance to tsetse fly species [43]. Altitude was also forced in the model to gain insights regarding the elevation limits of tsetse fly species distribution. Mean maximum temperature of the driest month was omitted from analysis because of the relatively lower knowledge of biological values of dryness to tsetse fly.

MaxeEnt was used to model the probability of species occurrence based on unique occurrence points [36-38]. Sample bias file was excluded in the model with the assumption that tsetse flies are likely to be present in large part of the study area due to widely spread of hosts [17; 48]. Because there were more than 15 occurrence points, MaxeEnt was run using linear, quadratic and hinge features [49]. The model was set to run with 500 iterations and 10 replicates with default parameters regularization and the jackknife estimates (measure of variable influence).

Model assessment

Four variables were included in MaxEnt along with the occurrence data. An initial SDM was run in MaxEnt (one run; raw output setting) to acquire lambda values used in ENMTools v.1.4. 3 [35] to calculate Akaike's Information Criterion (AICc; AIC and Bayesian information Criterion (BIC) [50] for a model fit with four, three and two variables, respectively (Table 2). This method selects the most parsimonious model. The model that was most parsimonious in this study (lowest AIC, AICc, BIC and high area under the receiver operating curve (AUC) value) had all four variables. The best model for each species was validated using 10-fold cross-validation, with the averages of 10 model runs representing the final output. Model performance as well as the contribution of predictor variables were assessed by using AUC and variable importance was assessed using the relative gain contribution of each variable and jackknife tests compared using

AUC, test gain and regularized training gain. Marginal and single variable response curve were used to depict the relationship between tsetse fly species and predictor variables. Final outputs included predictive maps of the probability of tsetse fly species presence based on climate suitability. The probability scores (numeric values between 0 and1) were displayed in ArcGIS 10.5 to show the current and future habitat suitability for each of the three tsetse fly species.

Results

Model selection

The distribution models for each tsetse fly species performed better than base/random (AUC>0.5). The model that included all four predictor variables had the best fit (Table 2). The results presented in all subsequent sections are based on this model.

Table 2: Model performance based on AUC, AIC, AICc and BIC values for tsetse fly species occurrence and different combinations of the environmental variables.

Species	Model assess ment	Tmax of warmest month Tmin of coldest month	Precipitation of the wettest month Tmax of warmest month Tmin of coldest month	Altitude Precipitation of the wettest month Tmax of warmest month Tmin of coldest month
G.m.morsitans	***AUC***	0.850	0.902	0.938
	AIC	702.78	667.27	625.79
	AICc	709.04	680.47	642.21
	BIC	714.51	683.39	643.38
G. pallidipes	***AUC***	0.818	0.919	0.959
	AIC	1302.59	1198.26	1108.75
	AICc	1304.79	1202.85	1115.54
	BIC	1317.13	1219.04	1133.68
G.swynnertoni	***AUC***	0.840	0.854	0.899
	AIC	624.99	614.66	576.83
	AICc	630.32	626.88	601.09

BIC	634.56	628.33	594.60

Variable contribution and climate suitability map for *G. m morsitans*

Altitude accounted for more than one third (35.1%) of the variation in climate suitability model for *G. m. morsitans* occurrence, followed by precipitation of the wettest month (32.1%), maximum temperature of the warmest month (22.3%), and minimum temperature of the coldest month (10.6%). Based on 10 percentile training presence logistic threshold (10% minimum threshold), the model showed that current suitable climate for *G.m.morsitans* covers 32% (19,225 km^2) of the entire Maasai Steppe (≈ 60,000 km^2) and in the future (year 2050) the model indicated the suitable area will shrink to 7.4% (4,447.34 km^2) of the current suitable area in the Maasai Steppe (Fig 2 and 3).

Variable response curves indicated that the probability of occurrence of *G.m.morsitans* drops off dramatically above 1000m of altitude when all variables are included in the model, but a very peaked response to altitude ≈1200msl and almost no probability of occurrence above 2500msl when that is the only variable considered (Supplementary 1 a and Supplementary 2 a). Marginal and single variable response curves were however similar for precipitation of the wettest month, showing a preference (probability of presence ≥ 0.6) for precipitation between 140-230mm per month, and almost no chance of occurrence below 100mm/month or above 350mm/month (Supplementary 1 b and Supplementary 2 b). The probability of occurrence of *G.m.morsitans* drops off dramatically above 28^0C maximum temperature when all variables are included in the model, but, a peaked response to maximum temperature of ≈ 28^0C for the mean maximum temperature of the warmest month, with minimal chances of occurrence below 15^0C

or above 32^0C maximum temperature values when used as the only single variable in the model (Supplementary 1 c and Supplementary 2 c).

The probability of occurrence of *G.m.morsitans* drops off dramatically above 14^0C minimum temperature when all variables are included in the model, reaching the peak response at a minimum temperature of ≈ 13^0C for the mean minimum temperature of the coldest month with rare chances of occurrence below 0^0C or above 16^0C minimum temperature when used as the only variable (Supplementary 1 d and Supplementary 2 d).

Variable contribution and climate suitability map for *G. pallidipes*

Precipitation of the wettest month accounted for almost two-third (60.4%) of the variation in habitat suitability, followed by altitude (23.0%) and maximum temperature of the warmest month (16.6%). Based on 10 percentile training presence logistic threshold, the model showed that current suitable habitat for *G.pallidipes* covers 11% (7113 km^2) of the Maasai Steppe and by 2050, the model indicated only 918 km^2 with suitable habitat for this species (Fig 4 and 5).

Variable response curves indicated that the probability of occurrence of *G.pallidipes* drops off dramatically above 1,000m of altitude when all variables are included in the model, reaching its peak response at altitude ≈1,200msl and almost no probability of occurrence above 3,000msl when that is the only variable considered in the model (Supplementary 3 a and Supplementary 4 a). Marginal and single variable response curves were similar for precipitation of the wettest month, showing a preference (probability of presence ≥ 0.6) for precipitation between 140-180ml per month, and almost no chance of occurrence below 120mm/month or above 330mm/month (Supplementary 3 b and Supplementary 4 b). The probability of occurrence of *G.pallidipes* drops

off dramatically above 28^0C maximum temperature when all variables are included in the model, and a peak response was observed at a maximum temperature of ≈ 28^0C for the mean maximum temperature of the warmest month, and almost no chance of occurrence below 10^0C or above 34^0C maximum temperature when used as the only variable (Supplementary 3 c and Supplementary 4 c). The probability of occurrence of *G.pallidipes* drops off dramatically above 10^0C minimum temperature when all variables are included in the model, but, a very peaked response to minimum temperature of ≈ 13^0C for the mean minimum temperature of the coldest month and almost no chance of occurrence below -5^0C or above 17^0C minimum temperature when used as the only variable (Supplementary 3 d and Supplementary 4 d).

Precipitation of the wettest month provided the best fit to the training data when used in isolation. This variable also appears to have the most information that is not present in the other variables, as it decreases the gain the most when it is omitted. Yet, precipitation of the wettest month indicated the best fit to the test data and best predicted the distribution of the *G. pallidipes* test data.

Variable contribution and climate suitability map for *G. swynnertoni*

Altitude contributed almost a half (47.5%) of the variation in climate suitability for *G. swynnertoni* occurrence, followed by precipitation of the wettest month (27.4%), minimum temperature of the coldest month (22%), and maximum temperature of the warmest month (3.1%). Based on 10 percentile training presence logistic threshold, it was revealed that, current suitable climate for *G. swynnertoni* covers 32,335 km^2, but is predicted to shrink to 7,374km^2 by the year 2,050 (Fig 6 and 7).

Variable response curves indicated that the probability of occurrence of *G.swynnertoni* drops off dramatically above 1000m of altitude when all variables are included in the model but a very peaked response to altitude ≈1300msl and almost no probability of occurrence above 2500msl when that is the only variable considered (Supplementary 5 a and Supplementary 6 a). Variable response curves indicated that the probability of occurrence of *G.swynnertoni* drops off dramatically above 140ml of rainfall when all variables are included in the model, but a very peaked response to precipitation ≈160ml for the precipitation of the wettest month and almost no probability of occurrence above 400ml/month or below 90ml/month when that is the only variable considered (Supplementary 5 b and Supplementary 6 b). The probability of occurrence of *G.swynnertoni* drops off dramatically above 28^0C maximum temperature when all variables are included in the model, but, a peaked response to maximum temperature of ≈ 28^0C for the mean maximum temperature of the warmest month, and almost no chance of occurrence below 10^0C or above 34^0C maximum temperature when used as the only variable (Supplementary 5 c and Supplementary 6 c).

The probability of occurrence of G.swynnertoni drops off dramatically above 14^0C minimum temperature when all variables are included in the model. The peak probability was observed at a minimum temperature of ≈ 14^0C for the mean minimum temperature of the coldest month with reduced chances of occurrence below 0^0C or above 16^0C minimum temperature when used as the only variable (Supplementary 5 d and Supplementary 6 d).

The best fit to the *G.swynnertoni* training data was provided by altitude when used by itself. Altitude indicated the best fit to the test data and best predicted the distribution of the *G swynnertoni* test data. Also, omission of this variable decreases the gain the most, meaning altitude had most information that is not present in other variables.

Discussion

Tsetse fly occurrence poses public health challenges and exacerbates economic hardships due to the investment needed to control tsetse flies and treat the diseases they transmit. Since climate is the dominant factor that determines tsetse fly occurrence, and the resources for controlling tsetse and trypanosomiasis are scarce, understanding how the changes in climate at local scale affects the spatial and temporal distribution of tsetse fly species is critical in identifying the most likely vulnerable places, and better targeting limited resources. The SDM used in this study provides useful information for public health, livestock development stakeholders and wildlife managers to plan for future potential climates effects across space and time.

This study used MaxEnt species distribution modelling to understand the influence of altitude and climate variables on tsetse fly species occurrence, and make predictions about future distribution based on predictive climate models. The models yielded current and future potential climate distribution maps for *G. m. morsitans, G.pallidipes and G. swynnertoni*, and predicted an overall reduction in the area of the Maasai Steppe that will have suitable climate for the three *Glossina* species. Prediction also indicated probability of these three tsetse fly species to inhabit relatively higher latitude by mid-century. Compared to current conditions, in the year 2050, area with suitable climate will decline to 23.13%, 12.9% and 22.8% of current suitable area for *G. m. morsitans, G.pallidipes* and *G.swynnertoni,* respectively. The reason for this could be explained by the temperature response curves, which indicated 34^0C mean maximum temperature of the warmest month and 17^0C mean minimum temperature of the coldest month to be maximum upper and lower temperature thresholds for these three species. The range reduction across the Maasai Steppe can be attributed to future climates exceeding these thresholds whereby, by mid of the

century, maximum temperature is expected to have risen by 1.7^{0}C in the Maasai Steppe [46]. The temperature thresholds that limit tsetse fly distribution and abundance has also been shown in other studies from the Maasai Steppe, based on intensive longitudinal sampling over smaller geographic areas [27]. These observations complement the suggestion that climate change in some parts of East Africa would result in overall reduction of habitat suitability range for tsetse flies, but also a spread out of suitable range particularly in high-altitude areas that currently are less suitable for the species due to low temperatures [18]. Hulme, also predicted a contraction of *G. m.morsitans* geographic range owing to climate change expected to affect the SADC region [51]. Influence of climate on the distribution of *Glossina species* has been explained in the previous studies [41, 42, 55] and *G. m.morsitans*, *G. pallidipes* and *G.swynnertoni* are among groups of tsetse flies whose relative abundance tends to decrease with high temperature. Our model forecasts suitable area for all three species that will shrink in the Maasai Steppe by 2050 under RCP 4.5, suggesting populations of these species may crash or may adapt to increasing maximum temperatures by moving upward in elevation. In fact, the models predicted a suitable altitude for *G. m.morsitans*, *G.pallidipes* and *G. swynnertoni* from around 1,000msl currently observed, to around 2,500m, 3,000m and 2,500m elevation, respectively, indicating these species may become problematic in high altitude ecosystems of the study area, if other ecological requirements for these species will be met in those habitats.

The importance of the four variables that were selected through our parsimony analysis to the ecology of the three *Glossina* species indicates the importance of careful scrutiny of available environmental data for a study site of interest. Although there was variation in variable contribution to specific species model, mean maximum temperature of the warmest month and mean minimum temperature for the coldest month indicated similar response curves. Specifically,

mean maximum temperature of the warmest month, and mean minimum temperature of the coldest month have relevant ecological importance to the distribution of tsetse fly species. For example, the logistic probability response curves indicated higher maximum temperature of the warmest month and higher minimum temperature of the coldest month decreases likelihood of all three *Glossina* species occurrence, likely because, both low and high temperatures affect development of all two tsetse species at various life stages [41]. Effects of hotter and colder environments on various developmental stages of tsetse fly species has also been reported [56-57].

Logistic probability response curves indicated that higher precipitation during the wettest month decreases the likelihood of occurrence of the three *Glossina* species considered in this study. Generally, no record is known on direct effect of rainfall on tsetse fly, but, it is thought that high rainfall may cause local flooding which may wash out pupae that are buried in loose soil, leading to tsetse fly depopulation and thus low probability of occurrence. Although responses to this variable indicated similar trend in all three species, the importance of the variable in models for the different species varied dramatically. For example, precipitation of wettest month contributing 60.4% of the relative gain to the *G. pallidipes* model and providing the best fit to the model, indicating that the species can respond differently to the climate variables. In particular, precipitation in the wettest month may be more important to the distribution of *G. pallidipes* owing to the species' ecology. *G. pallidipes* is strongly associated with wetter habitats, and so relatively hydrophilic, unlike *G.m.morsitans* and *G. swynnertoni.*

In all three tsetse fly species models, altitude had a relatively high contribution to the model gain, but did not necessarily provide the best fit to the training model. For example, altitude contributed 35.1% of relative gain to the *G.m. morsitans* model and 23% for *G. pallidipes*

respectively. However, the best fit to the training models for these two species were provided by mean maximum temperature of the warmest month and precipitation of the wettest month. This may be because temperature and rainfall have more biological relevance to tsetse flies compared to altitude. Although altitude indicated high contribution (47.5%) to the *G. swynnertoni* model and also had the best fit, it should however be noted that all occurrence points were obtained at a relatively lower altitudes and this might have influenced the results. Nevertheless, all *Glossina* species responded similarly to altitude, with response curves for all species indicating low preference for higher altitude. This is because higher altitudes are characterized by lower temperature that affects tsetse fly development [42]. Given that altitude and temperature were highly correlated, it was initially considered that by including altitude in the model, it could have masked the contribution of variables with greater biological relevance [37]. However, because relationships between tsetse flies and temperature are well-established [41,42,58,59], altitude was included in the models in order to gain insight into how tsetse fly species are likely to expand their range to higher elevations under future increases in temperature.

Extrapolated over larger areas, our findings could indicate either increases or decreases in suitable tsetse range. Likewise, predictions of climate impacts of tsetse distribution in Africa do not all agree. Some studies have suggested that climate change in some parts of East Africa would result in a spreading out of suitable range for tsetse flies particularly in high-altitude areas that currently exclude the species due to low temperatures, but also there is a chance of range contraction of tsetse flies in some location [18]. Other reports have suggested a decline in the distributional range of tsetse fly species owing to climate change. Furthermore, it should be noted that climate variables are not the sole predictors of future tsetse distribution. Other factors such as host availability and suitable vegetation will also influence where tsetse are found, but are more

difficult to model into the future. Distribution maps based on relationships with climate variables can therefore be considered to be maximum potential distributions.

Although the findings of this study are based on only a single GCM model, BCC-CSM1-1 from CMIP5, it is considered to have better predictive capacity because it uses RCP and at a relatively finer resolution of about 1km. The fact that these findings agree with previous findings reported by Rodgers and Randolph [50] and Hulme [51] that used relatively older GCM version, increase the confidence that climate is more likely to push distribution of tsetse flies into new areas, while removing it from others. For this reason, maps produced by this study can improve the efficiency and lower the cost of future surveillance. Also, the methods employed by this study can be adopted to generate high resolution species distribution maps under current and future climate scenarios for larger areas and for other vectors that pose threats to both public health and economic development. Tsetse fly control managers can incorporate the maps created from these models into integrated pest management regimes, and further tailor them based on what is already known about Maasai Steppe. Finally, maps such as these may be displayed to the public to increase awareness of climate change implications in the Maasai Steppe and other areas that are tsetse infested. These maps can as well inform protected areas managers of the likely encroachment due to shrinkage of tsetse fly habitats even in protected areas.

Limitation of this study include the fact that the study approach was climate envelope models which does not predict the expected ability/fitness of tsetse fly to adapt to the climate change. Inclusion of other ecological requirement variables would improve the prediction of general habitat suitability other than only climate suitability.

References

1. Meehl GA, Stocker TF, Collins WD, Friedlingstein P, Gaye AT, Gregory JM, Kitoh A, Knutti R, Murphy JM, Noda A, Raper SCB, Watterson IG, Weaver AJ, Zhao ZC. Global Climate Projections. In: Solomon S, Qin D, Manning M, Chen Z, Marquis M, Averyt KB, Tignor M, Miller HL, editors. Climate Change 2007: The Physical Science Basis. Contribution of Working Group I to the Fourth Assessment Report of the Intergovernmental Panel on Climate Change: 2007. pp. 747-846.

2. UNEP Fact sheet: Climate change in Africa - what is at stake? 2007 Dec 15 [Cited 2019 March 15] Available from: http://www.unep.org/roa/amcen/docs/AMCEN_Events/climate-change/2ndExtra_15Dec/FACT_SHEET_CC_Africa.pdf.

3. Davis MB, Shaw RG. Range shifts and adaptive responses to Quaternary climate change. Science. 2001 Apr 27 Vol292: 673-679. https://doi: 10.1126/science.292.5517.673

4. Thuiller W, Biodiversity: climate change and the ecologist. Nature. 2007 August 2Vol 448: 550-552. https://doi.org/10.1038/448550a

5. Thuiller W, Albert CH, Araújo MB, Berry PM, Cabeza M, Guisan G, Hickler T, Midgley GF, Paterson J, Schurr FM, Sykes MT, Zimmermann NE. Predicting global change impacts on plant species' distributions: Future challenges. Perspect. Plant. Ecol. Evol. Syst. 2008 Vol 9:137-152.

6. Konvicka M, Maradova M, Benes J, Fric Z, Kepka P. Uphill shifts in distribution of butterflies in the Czech Republic: Effects of changing climate detected on a regional scale. Glob. Ecol. Biogeogr. 2003 Vol 12: 403-410.

7. Franco AMA, Hill JK, Kitschke C, Collinggham YC, Roy DB, Fox R, Huntley B, Thomas CD.

Impacts of climate warming and habitat loss on extinctions at species' low- latitude range boundaries. Global Change Biology. 2006; 12 Vol 8:1545-1553. https://doi.org/10.1111/j.1365-2486.2006.01180.x

8. Hickling R, Roy DB, Hill JK, Fox R, Thomas CD (2006) The distributions of a wide range of taxonomic groups are expanding poleward. Glob. Chang. Biol. 2006 Vol 12:450-455. https://doi: 10.1111/j.1365-2486.2006.01116.x

9. Cook GC. Effect of global warming on the distribution of parasitic and other infectious diseases: a review. J. R. Soc. Med. 1992 Vol 85 (11): 688-691.

10. Gage KL, Burkot TR, Eisen, RJ, Hayes EB. Climate and Vectorborne Diseases. Am J Prev Med. 2008 Vol 35(5):436 – 450. https://doi: 10.1016/j.amepre.2008.08.030.

11. Mills JN, Gage KL, Khan AS. Potential Influence of Climate Change on Vector-Borne and Zoonotic Diseases: A Review and Proposed Research Plan. Environ. Health Perspect. 2010 Vol 118(11):1507–1514. https://doi: 10.1289/ehp.0901389

12. Moore S, Shrestha S, Kyle TW, Vuong H. Predicting the effect of climate change on African trypanosomiasis: integrating epidemiology with parasite and vector biology. J R Soc Interface. 2012 May 7 Vol 9 (70):817-30. https://doi: 10.1098/rsif.2011.0654.

13. Swallow BM. Impacts of trypanosomiasis on African agriculture. 1999 November 6 [Cited 2019 March 30]. Available from: http://citeseerx.ist.psu.edu/viewdoc/download?doi=10.1.1.631.6490&rep=rep1&type=pdf

14. Malele II. Fifty years of tsetse control in Tanzania : challenges and prospects for the

future. Tanzan J Health Res. 2010 Vol 13:1-10.

15. Sindato C, Kimbita EN, Kibona SN. Factors influencing individual and community participation in the control of tsetse flies and human African trypanosomiasis in Urambo District , Tanzania. Tanzan J Health Res. 2008 Vol 10(1): 20–27.

16. Malele I, Nyingilili H, Msangi A. Factors defining the distribution limit of tsetse infestation and the implication for livestock sector in Tanzania. Afr. J. Agric. Res. 2010 Vol 6(10): 2341–2347.

17. Daffa J, Byamungu M, Nsengwa G, Mwambembe E, Mleche W. Tsetse distribution in Tanzania: 2012 status. Tanzania Veterinary Journal. 2013 Vol 28:12-20.

18. Rogers DJ, Packer MJ. Vector-borne diseases, models, and global change. The Lancet 1993 Vol 342: 1282–1284.

19. Baker RHA, Sansford CE, Jarvis CH, Cannon RJC, MacLeod A, Walters KFA. The role of climatic mapping in predicting the potential geographical distribution of non-indigenous pests under current and future climates. Agric. Ecosyst. Environ. 2000 Vol 82(1–3):57–71.

20. Githeko AK, Lindsay SW, Confalonieri UE, Patz JA. Climate change and vector-borne diseases : a regional analysis. Bull. World Health Organ. 2000 Vol 78(9):1136-1347.

21. Watling JI, Romañach SS, Bucklin DN, Speroterra C, Brandt LA, Pearlstine LG, Mazzotti FJ. Do bioclimate variables improve performance of climate envelope models? Ecol. Model. 2012 Vol 246:79-85.

22. Watling JI, Brandt LA, Mazzotti FJ, Romañach SS. Use and Interpretation of Climate Envelope Models: A Practical Guide. University of Florida: 2013

23. Kearney MR, Wintle BA, Porter WP. Correlative and mechanistic models of species distribution provide congruent forecasts under climate change. Conserv Lett. 2010 Vol 3:203-213.

24. Malele II, Kibone SN, Matemba LE, Sahani K, Swilla J, Mwalimu CD, Mayala BK, Kimaro E, Msumary C, Kalinga RB. Human African trypanosomiasis and challenges to its control in Urambo, Kasulu and Kibondo Districts, western Tanzania. Tanzan J Health Res. 2006 Vol 8: 80-85.

25. Sindato C, Malele I, Mwalimu C, Myingilili HS, Kaboya S, Kombe E, Msumary C, Manoza A. Seasonal variation in human African trypanosomiasis in Tarangire National Park in Babati District, Tanzania . Tanzan Health Res Bull. 2007 Vol 9(2):2005–2008.

26. Salekwa LP, Nnko HJ, Ngonyoka A, Estes AB, Agaba M, Gwakisa PS. Relative abundance of tsetse fly species and their infection rates in Simanjiro, Northern Tanzania. Livestock Research for Rural Development 2014 Vol 26(12):1–8.

27. Nnko HJ, Ngonyoka A, Salekwa PL, Estes BA, Hudson PJ, Gwakisa PS, Cattadori IM. Seasonal variation of tsetse fly species abundance and prevalence of trypanosomes in the Maasai Steppe, Tanzania. J Vector Ecol. 2017 Vol 42(1):24-33. https://doi: 10.1111/jvec.12236.

28. Nnko, HJ, Gwakisa, PS, Ngonyoka A, Saigilu M, Ole-Neselle M, Kisoka W, Sindato C, Estes BA. Pastoralists’ vulnerability to trypanosomiasis in MaasaiSteppe. EcoHealth. 2017 November 02 Vol 14(4):718-731. https://doi:10.1007/s10393-017-1275-4 29. Ngonyoka A, Gwakisa SP, Estes BA, Nnko JH, Hudson JP, Cattadori MI. Variation of tsetse fly abundance in relation to habitat and host presence in the Maasai Steppe, Tanzania. J Vector Ecol. 2017 Vol 42: 34-

43.https:// doi: 10.1111/jvec.12237

30. Ngonyoka A, Gwakisa SP, Estes BA, Salekwa PL, Nnko, JH, Hudson JP, Cattadori MI. Patterns of tsetse abundance and trypanosome infection rates among habitats of surveyed villages in Maasai steppe of northern Tanzania. Infect Dis Poverty.2017 September 4 6(1):126. doi: 10.1186/s40249-017-0340-0.

31. Ngongolo K, Estes AB, Hudson PJ, Gwakisa PS. Influence of Seasonal Cattle Movement on Prevalence of Trypanosome Infections in Cattle in the Maasai Steppe, Tanzania. J Infect Dis Epidemiol 2019 Vol 5:079. doi.org/10.23937/2474-3658/1510079

32. Kshatriya M, Kifugo S, Msoffe F, Neselle M, Said MY (2007). Novel Forms of Livestock and Wildlife Integration Adjacent To Protected Areas in Africa – Tanzania; Mapping Land Cover Changes in Simanjiro and Monduli Districts. International Livestock Research Institute. FAO Report on GEF Tanzania project. Rome (Italy): FAO 2002.

33. Hargrove JW, Holloway MTP, Vale GA, Gough AJE. Catches of tsetse (*Glossina* spp.) (Diptera: Glossinidae) from traps and targets baited with large doses of natural and synthetic host odour. Bull Entomol Res.1995 Vol 85: 215-227.

34. Bouyer J, Seck TM, Sall B, Ndiaye YE, Guerrini L, Vreysen JBM. Stratified Entomological Sampling in Preparation for an Area-Wide Integrated Pest Management Program: The Example of Glossina palpalis gambiensis (Diptera: Glossinidae) in the Niayes of Senegal, J. Med. Entomol. 2010 July 01 Vol 47(4):543–552.

35. Warren DL, Glor RE, Turelli M. ENMTools: a toolbox for comparative studies of environmental niche models, Ecography 2010 July 28 Vol 33: 607-611.

https://doi.org/10.1111/j.1600-0587.2009.06142.x

36. Phillips SJ, Anderson RP, Schapire RE. Maximum entropy modeling of species geographic distributions, Ecol. Model.2006 Vol 190: 231–259.

37. Elith J, Phillips SJ, Hastie T, Dudík M, Chee YE, Yates CJ. A statistical explanation of MaxEnt for ecologists. Diversity Distrib. 2011 Vol 17:43–57. https://doi.org/10.1111/j.1472-4642.2010.00725.x

38. Phillips SJ, Dudik M, Elith J, Graham CH, Lehmann A, Leathwick J, Ferrier S. Sample selection bias and presence-only distribution models: implications for background and pseudo-absence data. Ecol Appl. 2009 Vol 19: 181-197. https://doi: 10.1890/07-2153.1

39. Peterson AT, Soberón J, Pearson RG, Anderson RP, Martínez-Meyer E, Nakamura M, Araújo MB. Species' Occurrence Data. In: Levin SA, Horn HS editors. Ecological niches and geographic distributions. Princeton University Press, Princeton and Oxford. 2011.pp. 62-180.

40. Bradley BA. Predicting abundance with presence-only models, Landsc. Ecol.2016 Vol 31(1):19–30. https://doi.org/10.1007/s10980-015-0303-4

41. Hargrove JW. Factors affecting density-independent survival of an island population of tsetse flies in Zimbabwe, Entomol Exp Appl 2001 Vol 100(2):151–164.

42. Hargrove JW. Tsetse population dynamics. In: The Trypanosomiases. Maudlin I, Holmes PH, MA editors. 2004 pp. 113-138.

43. Van Den Bossche P, De Deken R. Seasonal variations in the distribution and abundance of the tsetse fly, Glossina morsitans morsitans in eastern Zambia. Med. Vet. Entomol. 2002 June 01 Vol 16(2):170–176. https://doi: 10.1046/j.1365-2915.2002.00360.x

44. Lukaw YS, Abdelrahman MM, Mohammed YO, Ochi EB, Elrayah IE. Factors influencing seasonal abundance of *Glossina fuscipes fuscipes* (Glossina: Glossinidae) in KajoKeji County, South Sudan, Curr. Res. J. Biol. Sci.2014 Vol 6: 222-228.

45. Pollock JN. Training Manual for Tsetse Control Personnel:Volume 2, Ecology and Behaviour of Tsetse. FAO, Rome; 1982.

46. Jack C, Kloppers P. Provision of climate projections and analysis For the WHO/TDR Project B20280 "Predicting vulnerability and improving resilience of the Maasai communities to vector borne infections: an Eco-health approach in the Maasai Steppe ecosystem" University of Cape Town, South Africa.2016.

47. Hijmans RJ, Elith J. Species distribution modeling with R Introduction. 2017 January 08 [Cited 2018 April 22]. Available from: http://cran.rproject.org/web/packages/dismo/vignettes/dm.pdf.

48. Msoffe FU, Said MY, Ogutu JO, Kifugo SC, De JG, Paul VR, Robin S. Spatial correlates of land-use changes in the Maasai- Steppe of Tanzania : Implications for conservation and environmental planning, Int. J. Biodivers. Conserv. 2011 Vol 3:280-290.

49. Bateman BL, Vanderwal J, Johnson CN.. Nice weather for bettongs: Using weather events, not climate means, in species distribution models. Ecography. 2012 May 03 Vol 35(4):306–314. https://doi.org/10.1111/j.1600-0587.2011.06871.x

50. Anderson D, Burnham K. Avoiding pitfalls when using information-theoretic methods, J Wildl Manage: 2002 Vol 66(3): 912–918.

51. Hulme M. Climate Change and Southern Africa. An Exploration of Some Potential Impacts and Implications in the SADC Region. WWF International Climate Research Unit, Norwich.1996.

52. Rogers DJ, Randolph SE. Distribution and abundance of tsetse flies (Glossina spp.), J Anim Ecol.1986 Vol 55:1007–1025.

53. Rogers BDJ, Ki SIY, Packer MJ. Predicting the distribution of tsetse flies in West Africa using temporal Fourier processed meteorological satellite data, Ann. Trop. Med. Parasitol. 1996 Vol 90: 225–241.

54. Moore N, Messina J. A landscape and climate data logistic model of tsetse distribution in Kenya, PloS one 2010 Vol 5(7) e11809. https://doi:10.1371/ journal.pone.0011809.

55. Albert M, Wardrop NA, Atkinson PM, Torr SJ, Welburn SC. Tsetse Fly (*G.f. fuscipes*) Distribution in the Lake Victoria Basin of Uganda, PLOS Negl. Trop. Dis. 2015 Vol 9(4) e0003705. https://doi:10.1371/ journal.pntd.0003705

56. Torr SJ, Hargrove JW. Behaviour of tsetse (Diptera: Glossinidae) during the hot season in Zimbabwe: the interaction of micro-climate and reproductive status, Bull Entomol Res. 1999 Vol 89(4):365–379.

57. Terblanche JS, Clusella-Trullas S, Deere JA, Chown SL. Thermal tolerance in a south-east African population of the tsetse fly Glossina pallidipes (Diptera, Glossinidae): implications for forecasting climate change impacts, J Insect Physiol. 2008 Vol 54(1):114–27.

58. Rogers DJ. A general model for the African trypanosomiases. J. Parasitol. 1988 Vol 97 (1):

193–212.

59. Hargrove JW. The effect of ambient temperature on the flight performance of the mature male tsetse fly, Glossina morsitans, Physiol Entomol. 1980 Vol 5:397–400.

Supporting information Captions

Figures

Fig 1: An extract of map of Tanzania showing the study districts (Kiteto, Longido, Monduli and Simanjiro) forming the Maasai Steppe.

Fig 2 Current climate suitability maps for the best performing model with the *G.m.morsitans* occurrence data, and all 4 environmental variables: elevation, precipitation of the wettest month (April), mean maximum temperature of the warmest month (February), and mean minimum temperature of the coldest month (July).

Fig 3: Midcentury (2050) climate suitability maps for the best performing model with the G.m.morsitans occurrence data, and all 4 environmental variables: elevation, precipitation of the wettest month (April), mean maximum temperature of the warmest month (February), and mean minimum temperature of the coldest month (July). In these figure we see that the probability of occurrence decreases with time (comparing current and midcentury) from the maximum values of 0.845 to 0.658, with contracted habitat

Fig 4: Current climate suitability map for the best performing model with the G.pallidipes occurrence data, including all 4 variables.

Fig 5: Midcentury (2050) climate suitability map for the best performing model with the G.pallidipes occurrence data, including all 4 variables. In these maps we see that the probability of occurrence decreases with time (comparing current and midcentury) from the maximum values of 0.919 to 0.725, with shrunk habitat

Fig 6: Current climate suitability maps for G. swynnertoni, for the model including all four predictor variables.

Fig 7: Midcentury (2050) climate suitability maps for G. swynnertoni, for the model including all four predictor variables. Similarly, in these maps indicate that the probability of occurrence decreases with time (comparing current and midcentury) from the maximum values of 0.826 to 0.715, with narrowing habitat

Supplementary

Supplementary 1: a-d shows marginal response curves for the best performing model with *G.m.morsitans* occurrence data. Temperature is reported in 0C * 10.

Supplementary 2: a-d shows single variable response curves for the best performing model with *G.m.morsitans* occurrence data. Temperature is reported in 0C * 10.

Supplementary 3: a-d shows marginal response curves for the best performing model with *G.pallidipes* occurrence data. Temperature is reported in ^{0}C * 10.

Supplementary 4: a-d shows single variable response curves for the best performing model with *G.pallidipes* occurrence data. Temperature is reported in ^{0}C * 10.

Supplementary 5: a-d shows marginal response curves for the best performing model with G.*swynnertoni* occurrence data. Temperature is reported in ^{0}C * 10.

Supplementary 6: a-d single variable response curves for the best performing model with G.*swynnertoni* occurrence data. Temperature is reported in ^{0}C * 10.

Click here to download Figure Figure 1_Study site.tif

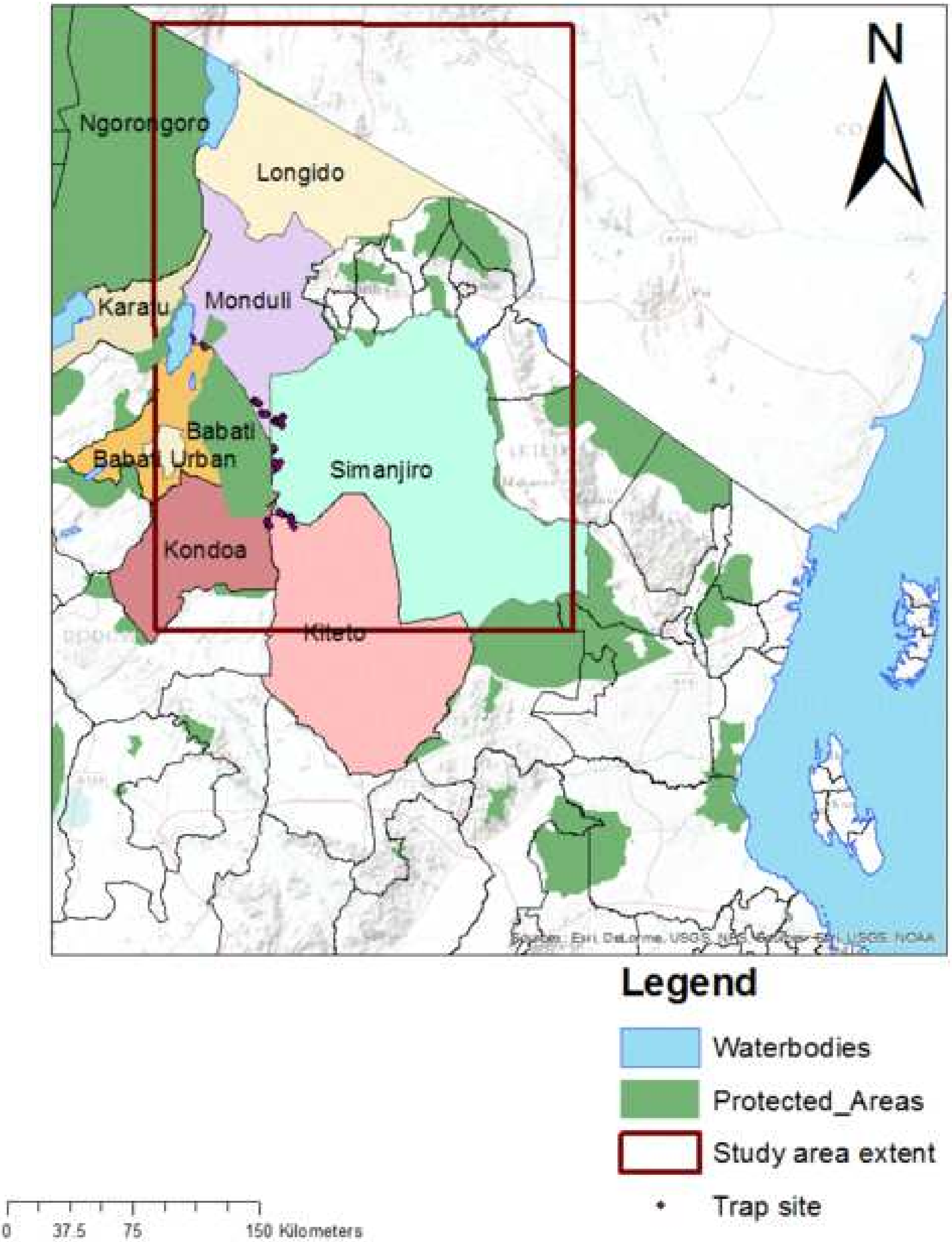

Click here to download Figure Figure 2_G Morsitans Current_.tif

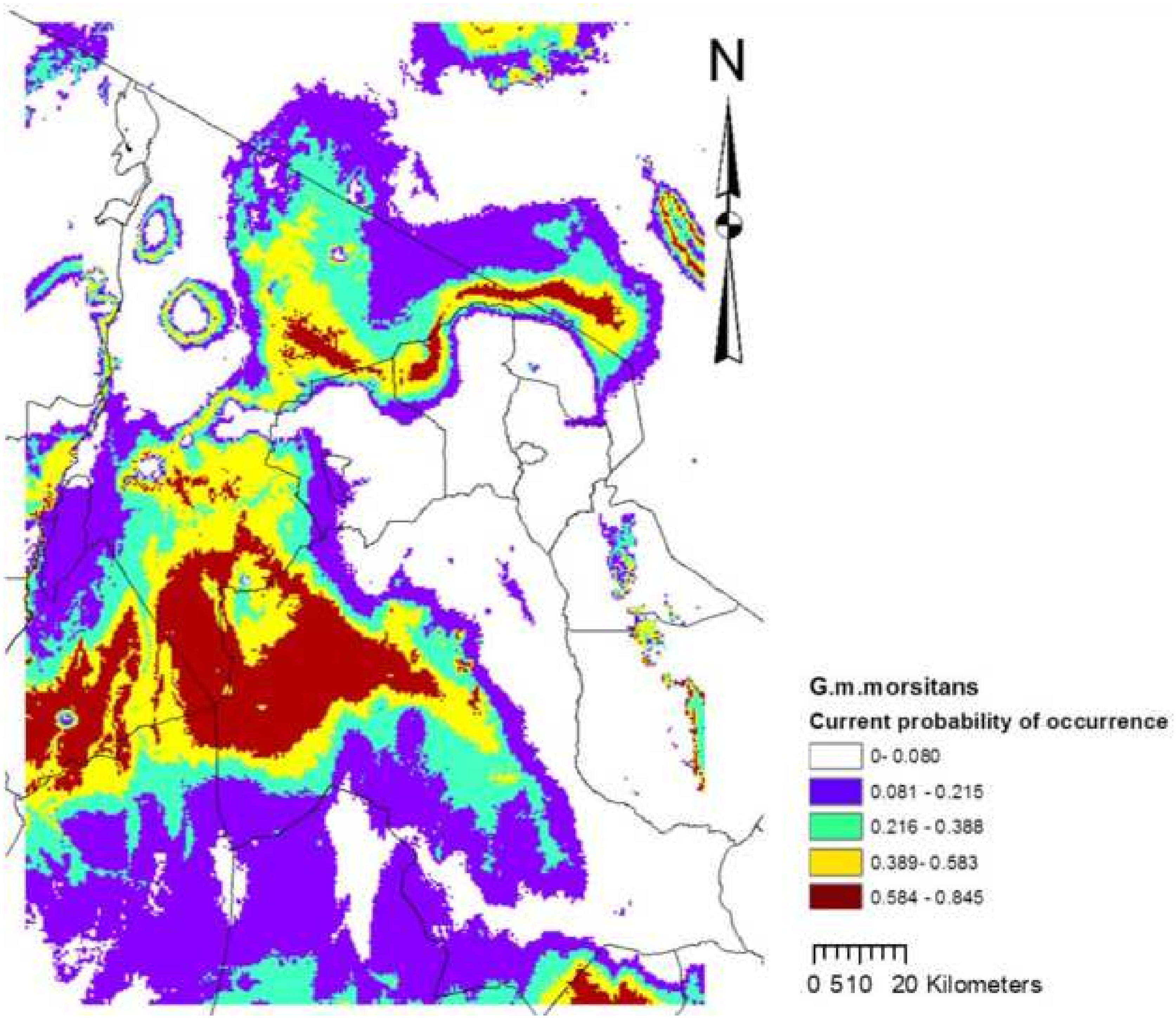

Click here to download Figure Figure 3_ G Morsitans Future _.tif

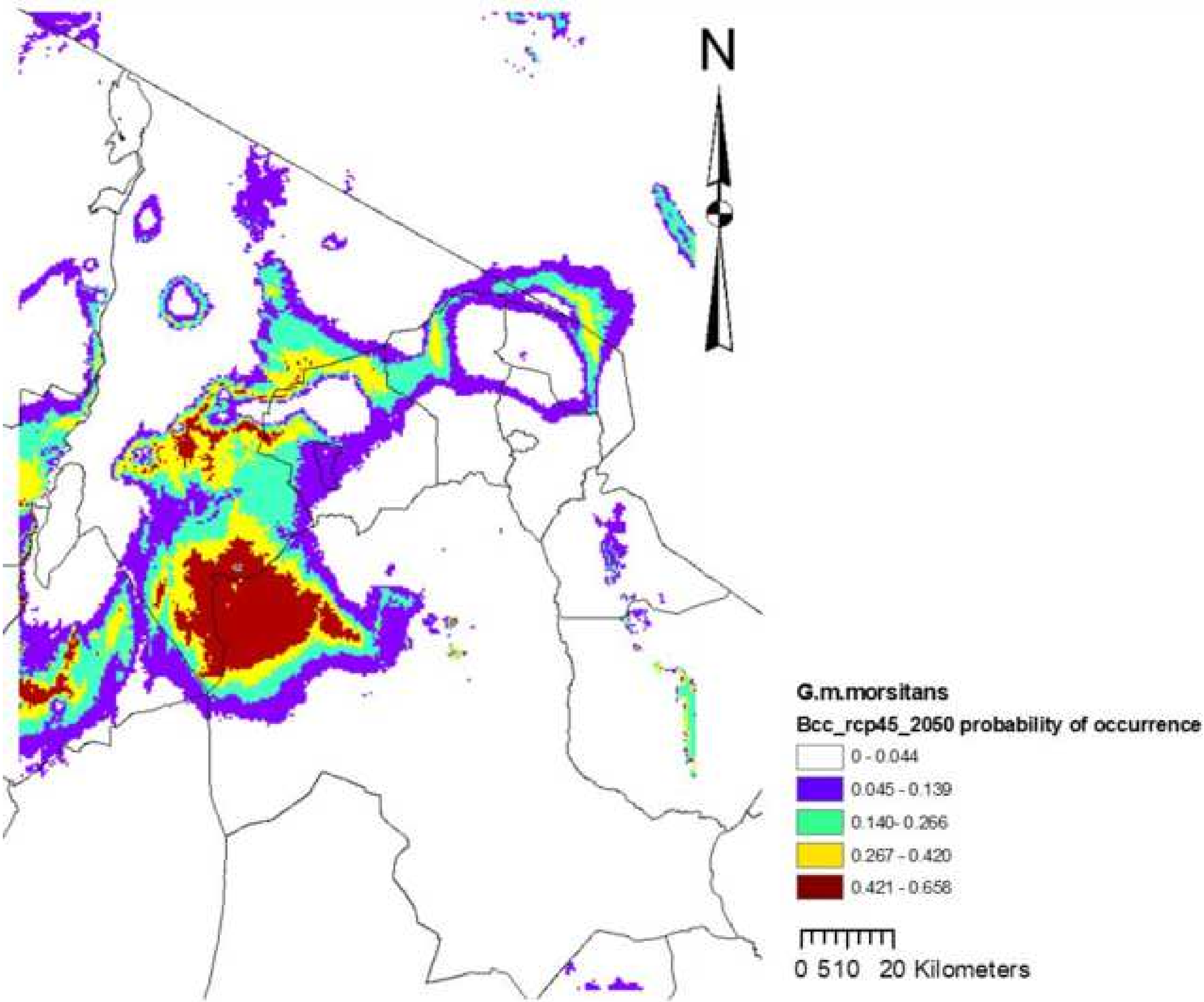

Click here to download Figure Figure 4_Pallidipes Current_.tif

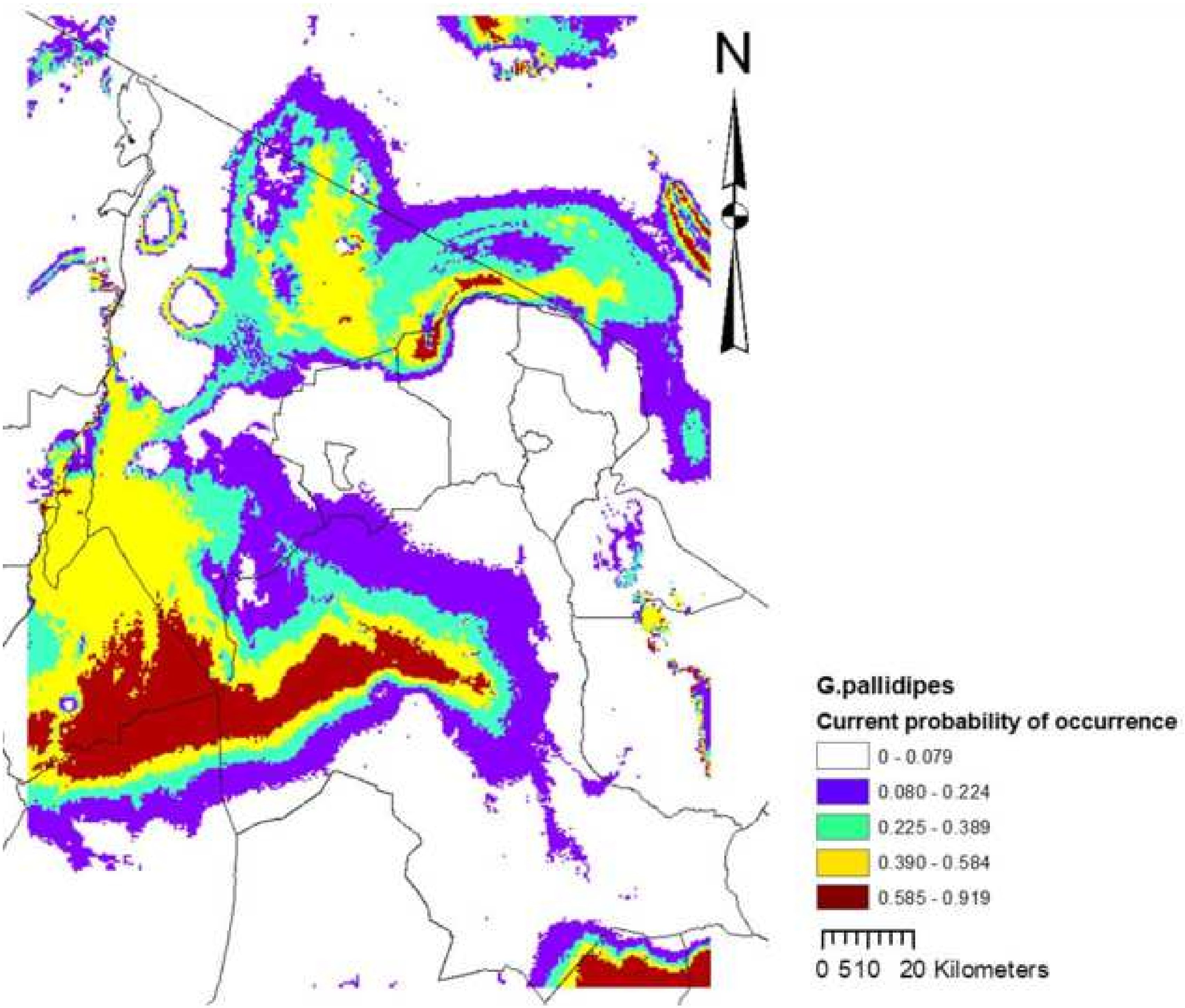

Click here to download Figure Figure 5_Pallidipes Future_.tif

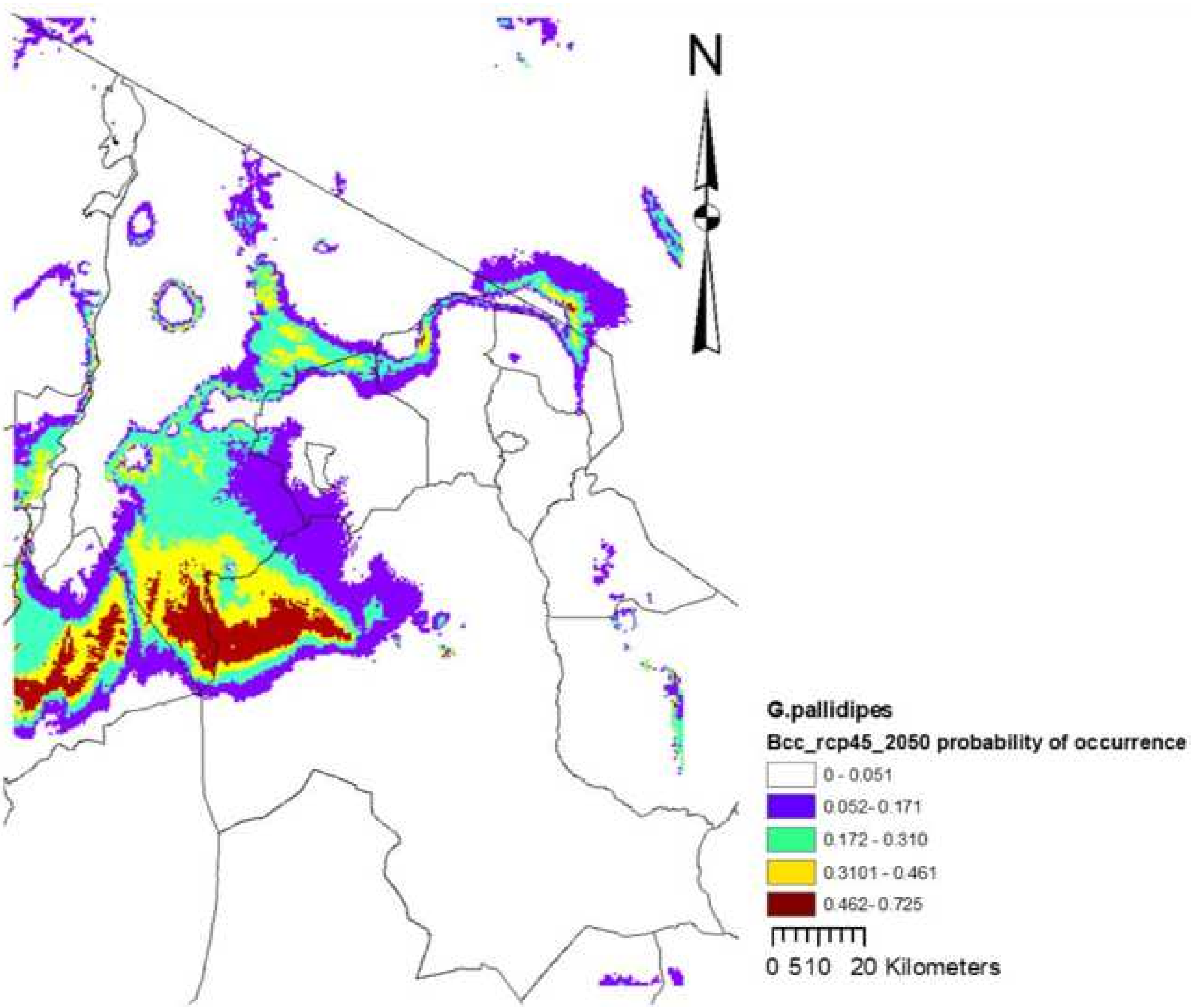

Click here to download Figure Figure 6_ Swynnertni Current_.tif

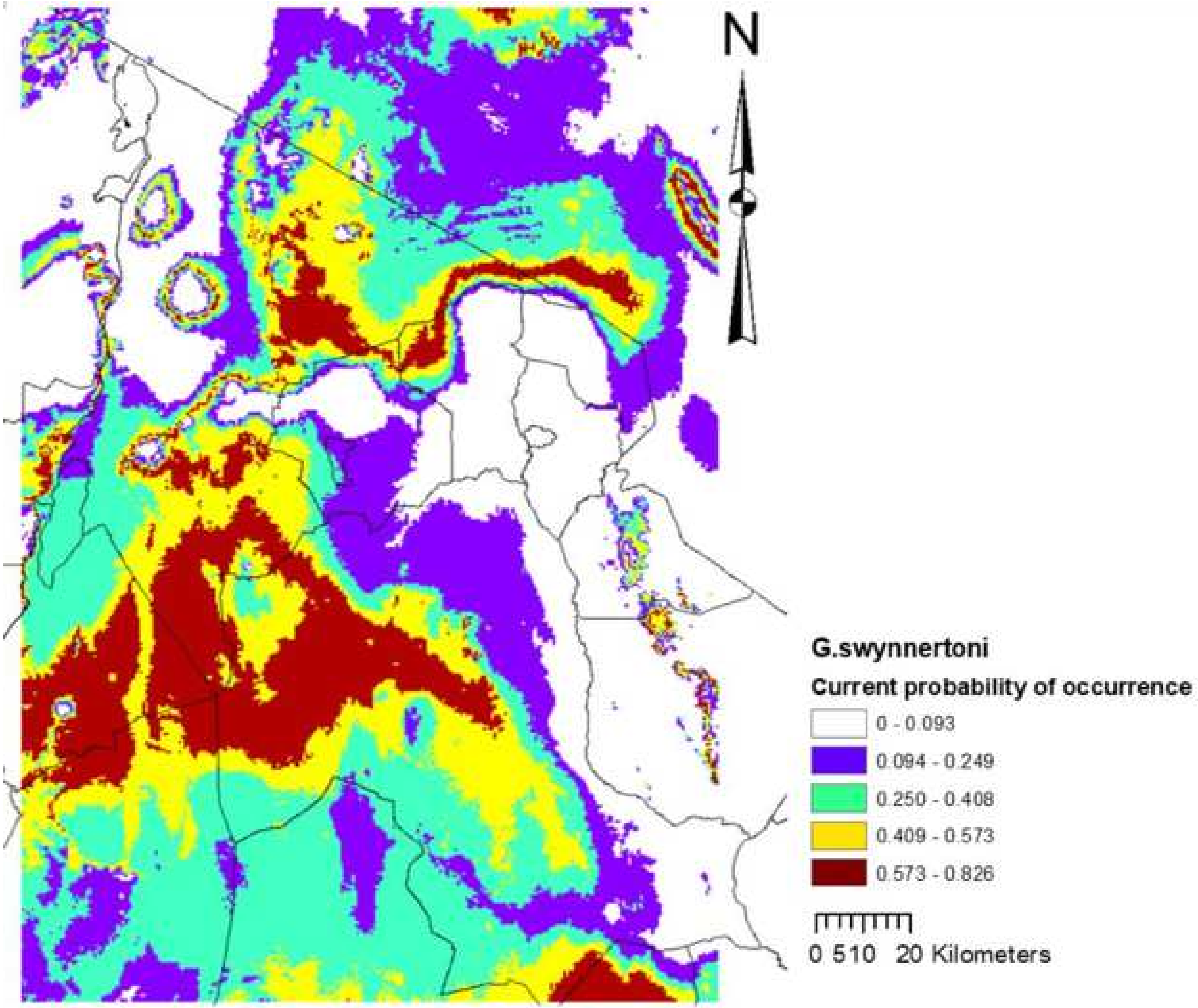

Click here to download Figure Figure 7_ Swynnertni Future_.tif

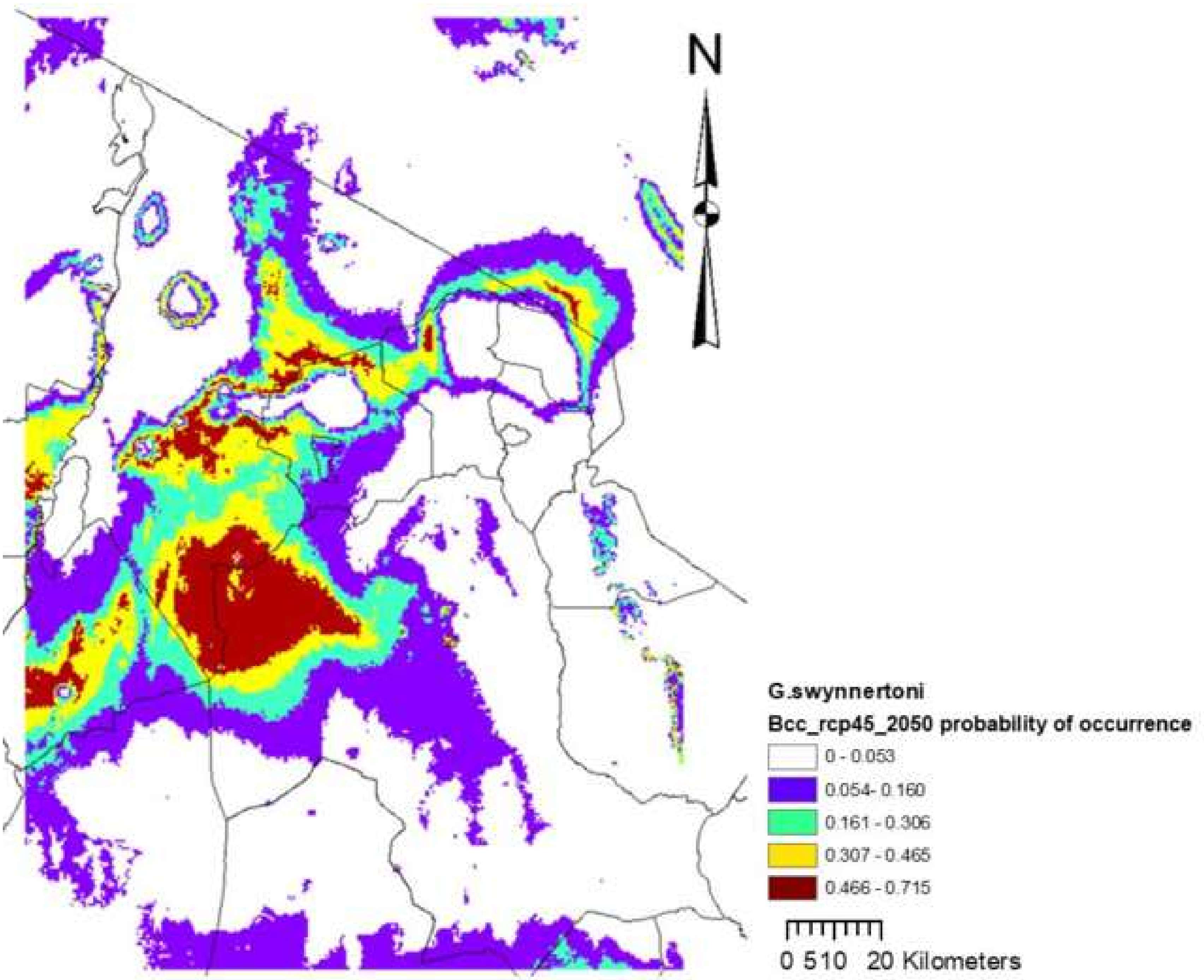

Click here to download Figure Supplementary 1.tif

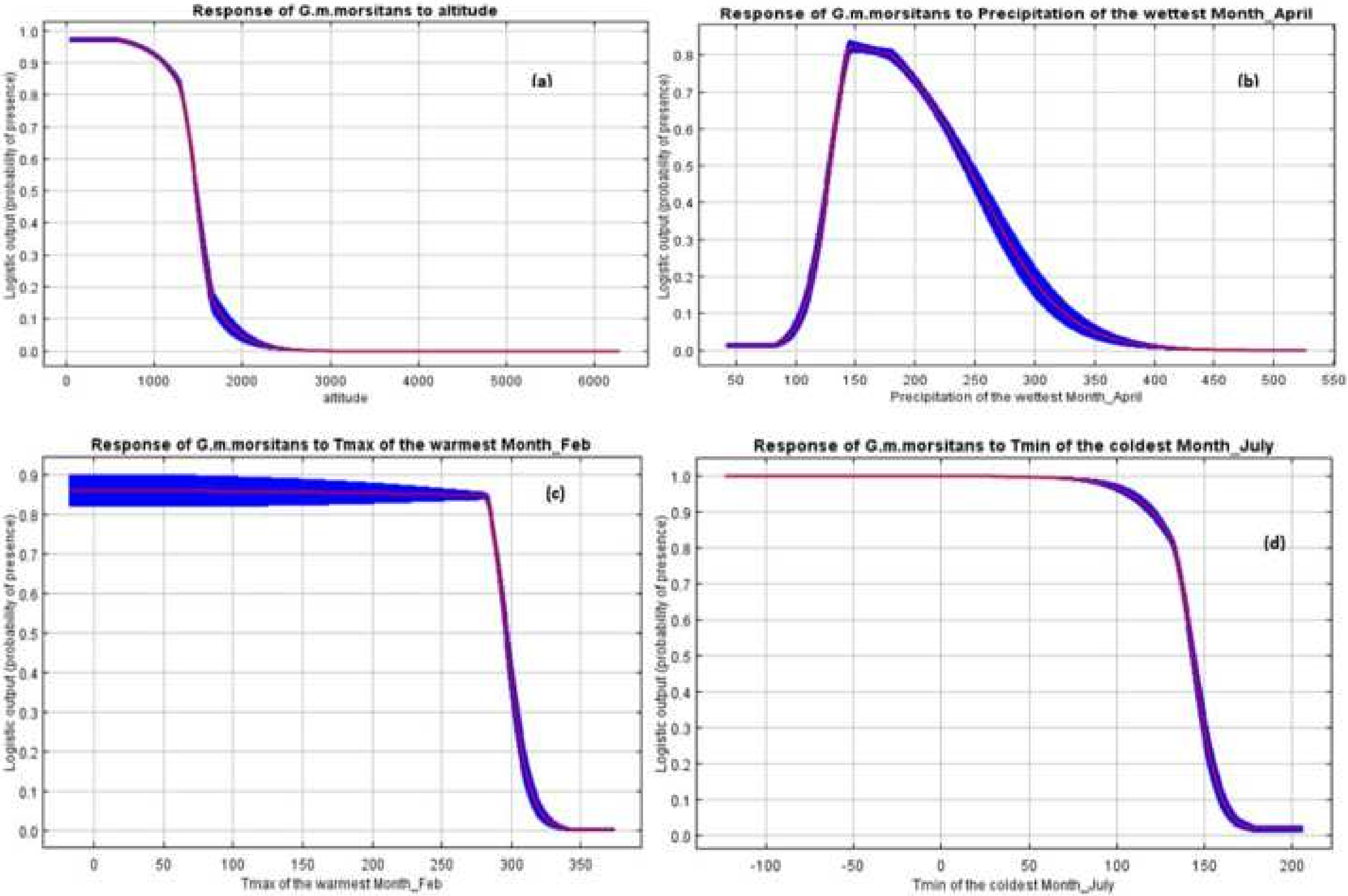

Click here to download Figure Supplementary 2.tif

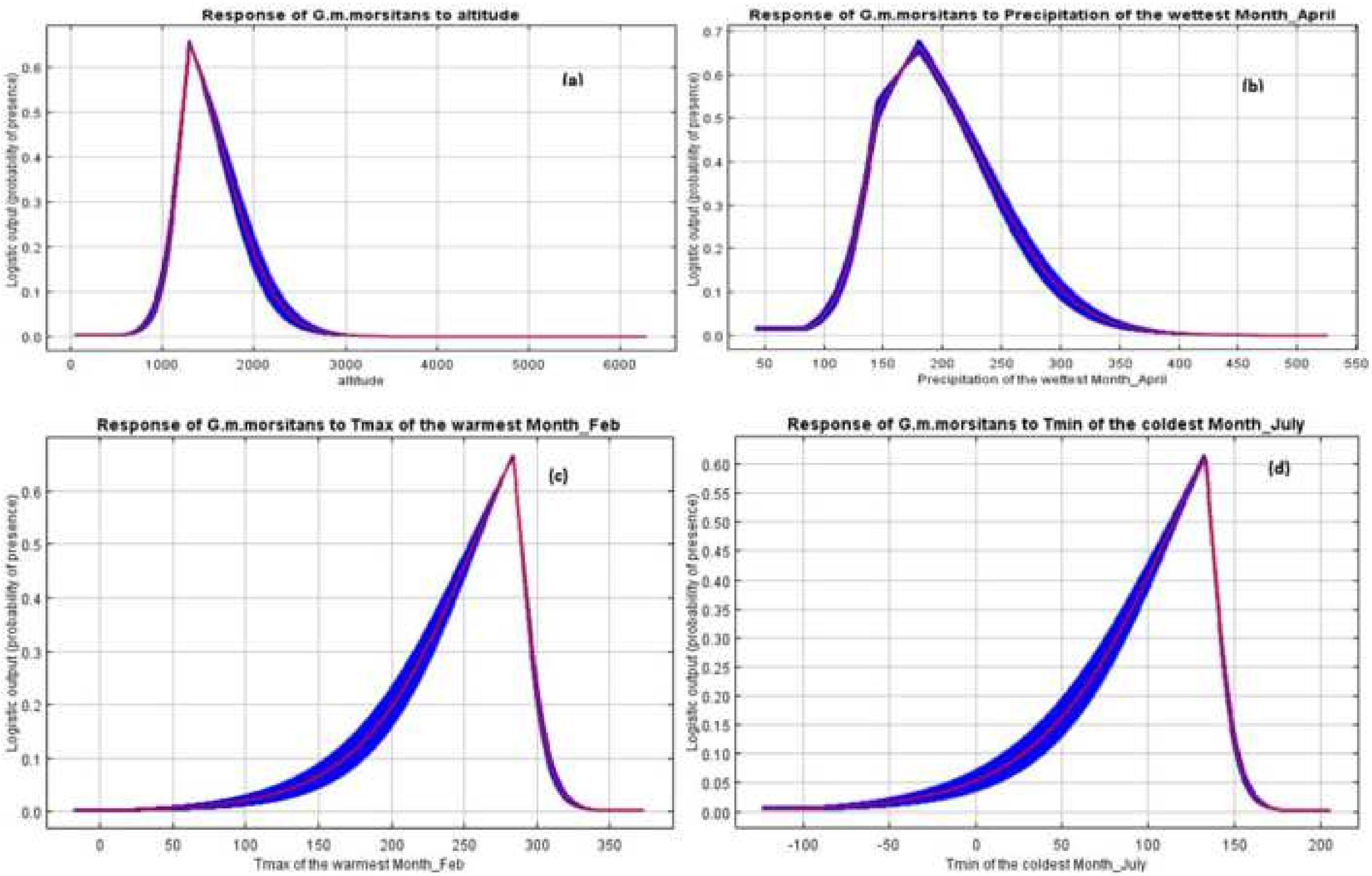

Click here to download Figure Supplementary 3.tif

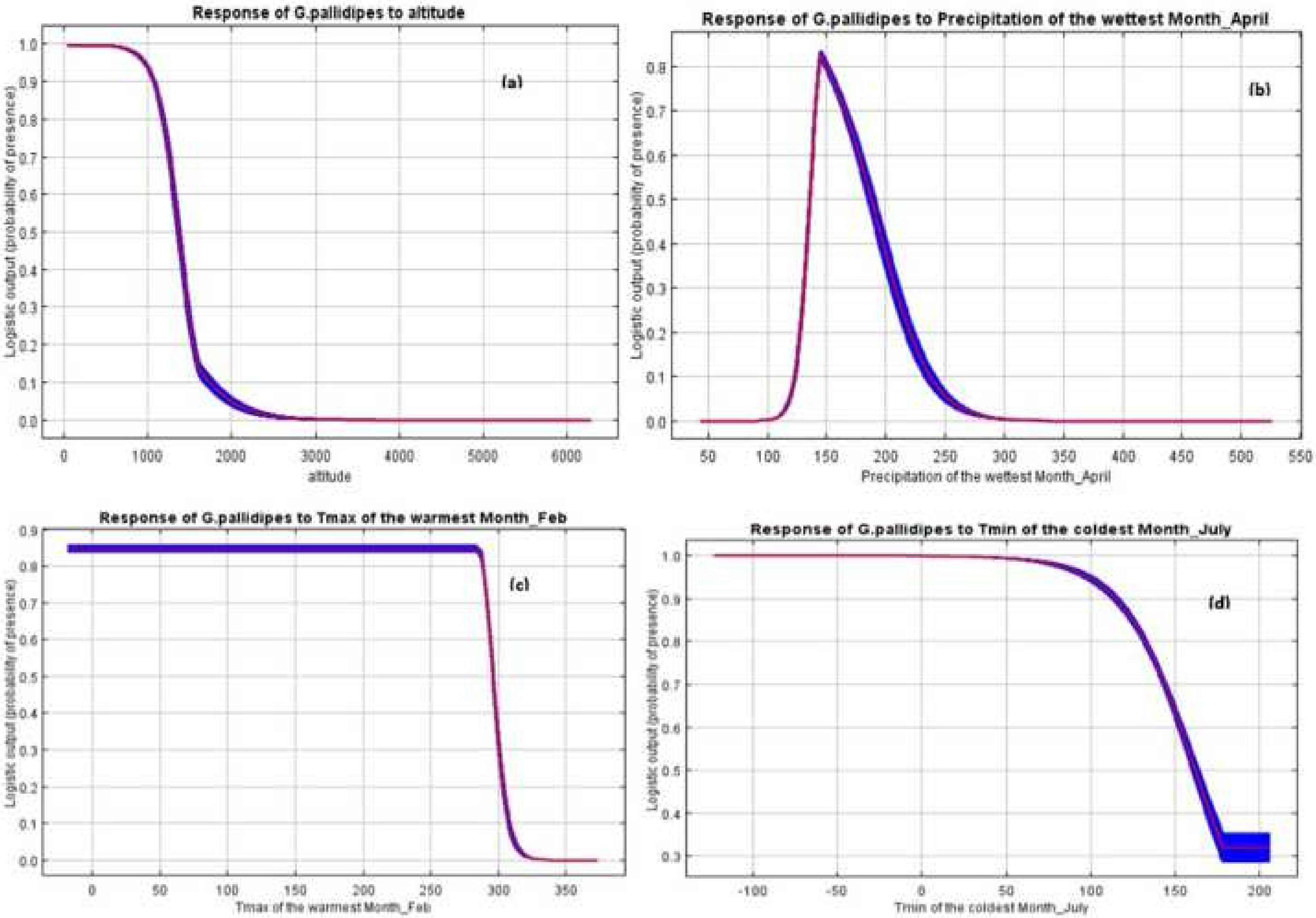

Click here to download Figure Supplementary 4.tif

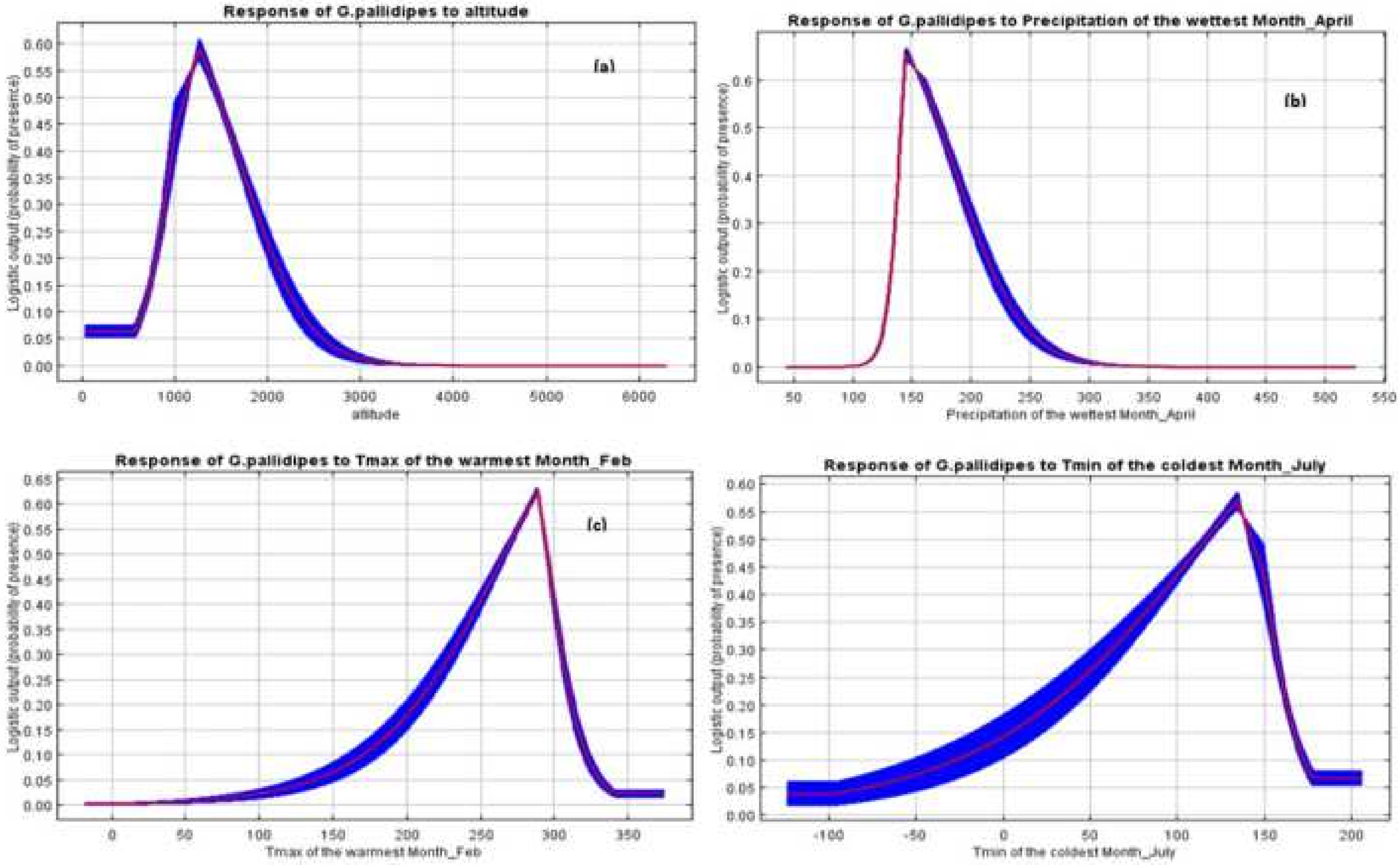

Click here to download Figure Supplementary 5.tif

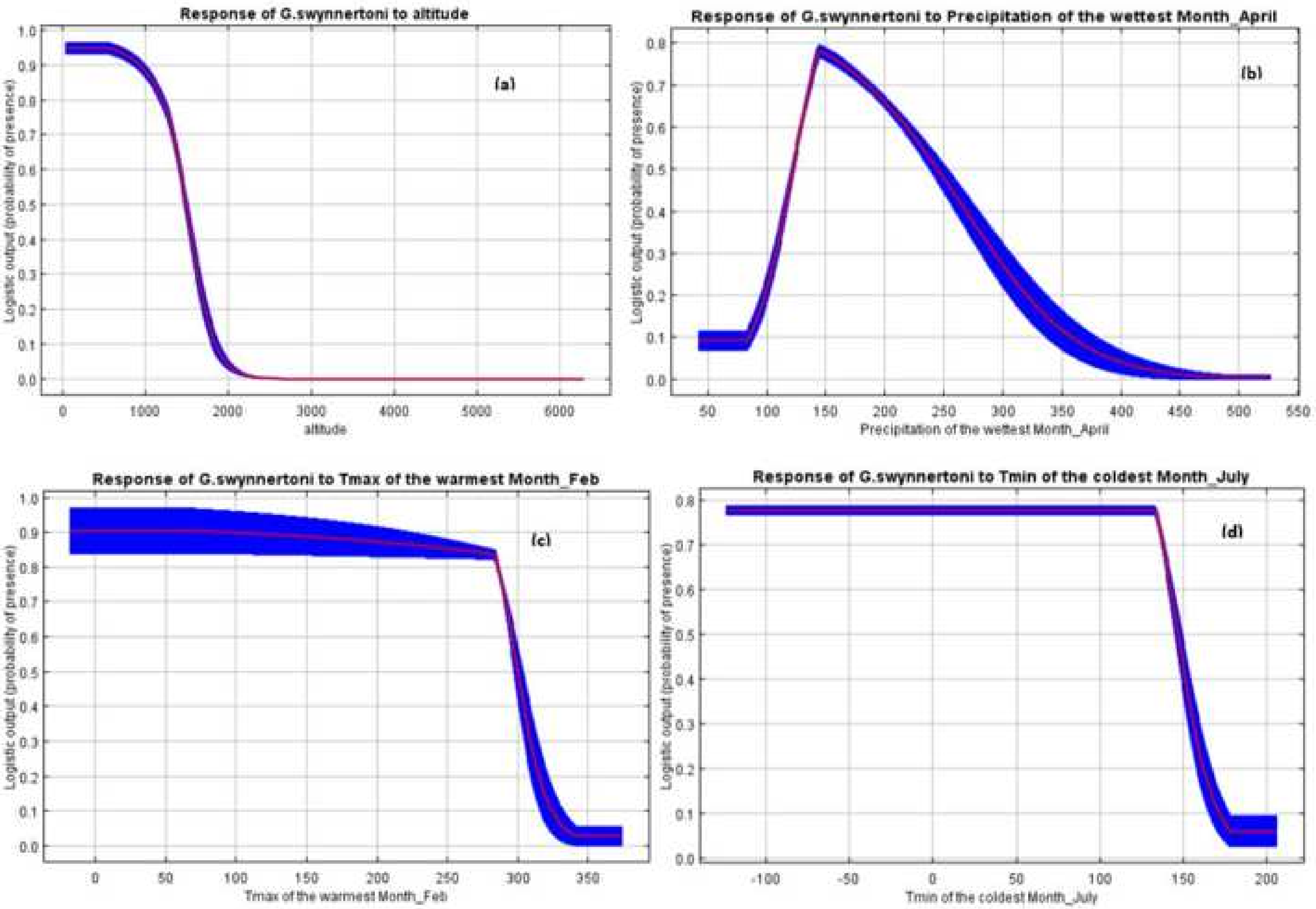

Click here to download Figure Supplementary 6.tif

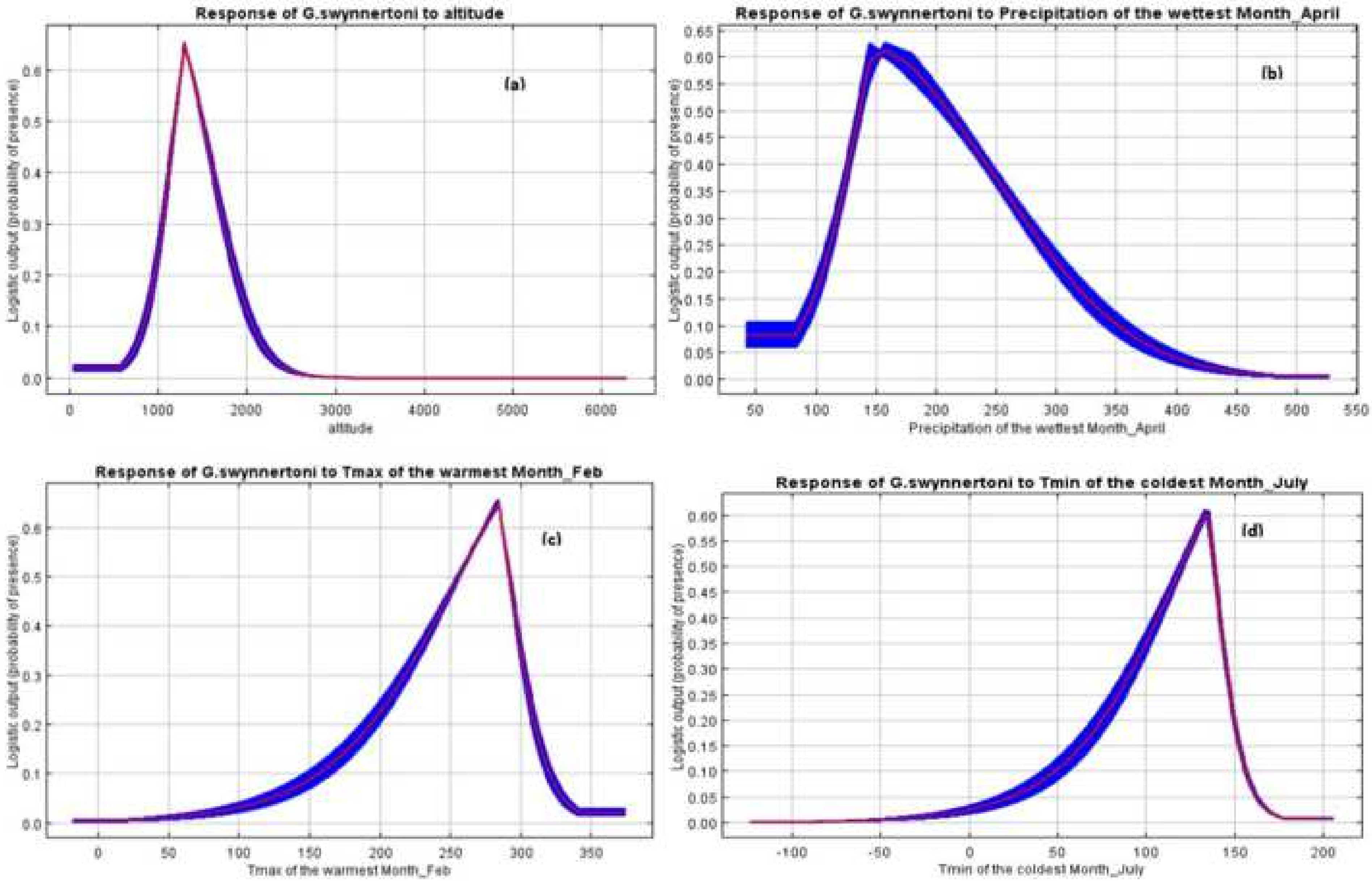

Full title: **Potential Impacts of Climate Change on Geographical Distribution of Three Primary Vectors of African Trypanosomiasis in Tanzania Maasai Steppe; *G. m. morsitans, G. pallidipes and G. swynnertoni***

Short title: **Climate Change and Vectors of African Trypanosomiasis**
