# Supplementary material for: Potential impacts of climate change on geographical distribution of three primary vectors of African Trypanosomiasis in Tanzania’s Maasai Steppe: G. m. morsitans, G. pallidipes and G. swynnertoni
Source: PLoS Negl Trop Dis. 2021 Feb 11;15(2):e0009081. doi: 10.1371/journal.pntd.0009081 (PMC7904224; doi:10.1371/journal.pntd.0009081)
Supplement: Attachment — Submitted filename: Nnko_Response to the Review Comments_28 September 2020.doc [file pntd.0009081.s012.doc]

# Response to the Reviewers’ Comments

***Journal:***	PLOS Neglected Tropical Diseases

***Manuscript ID:***	PNTD-D-20-00736

***Title:***	 Potential Impacts of Climate Change on Geographical Distribution of Three Primary Vectors of African Trypanosomiasis in Tanzania Maasai Steppe; *G. m. morsitans, G. pallidipes* and *G. swynnertoni*

***Corresponding***

***Author’s e-mail:***	happyjackson.nnko@gmail.com

***Date:***	28 September 2020

The authors would like to thank the reviewers for taking their time to give such detailed comments, which we feel have substantially improved the manuscript. Please note that after revisions, the original page and line numbers used by the reviewers may no longer match the text of the manuscript, but we have referred to line numbers where the changes have been made in our responses and all changes made are highlighted yellow and green for convenience.

**RESPONSES TO REVIEWERS’ COMMENTS**

**A: GENERAL COMMENTS**

Reviewer #1:

*The study appears to have been well conducted. However, it requires significant editorial correction or improvement, and specially the Discussion part, which appears to be too wordy and thematically repetitive.*

RESPONSE

This is an important observation and indeed editorial correction has been done and aspects of repetition has been corrected**.**

Reviewer #2:

*The objective of the study is clearly articulated with a clear testable hypothesis stated and the study design is appropriate, population described. The statistical analysis supports the conclusion, but use of a single wet season and a single dry season may not be enough to make proper conclusions for such a study. One season may not give representative data. It would be good if they could consider data for atleast 3 wet seasons and 3 dry seasons.*

RESPONSE

Reviewers might be right to some extent and actually having more data as suggested could have been particularly important in understanding the ecology and affirmation of seasonal population dynamics. However, aim of this study was to use current and future climate data and tsetse fly current occurrence data to predict current and future probability of tsetse fly occurrence in entire areas of Maasai Steppe (including areas where samples have not been collected) and this is how MaxENT works. In addition, and from previous studies, we already know the potential distribution of tsetse fly in dry and wet season, thus we are sure that season repetition would not bring significant changes as we would be sampling on the same area.

Reviewer #2:

*I have suggested published articles of previous work in this area for the authors to read before making certain statements, details are in the manuscript (attached). These are minor revisions in the abstract and methods.*

RESPONSE

This is an important observation and indeed comments have been incorporated as indicated in specific comments**.**

Reviewer #2:

*The work is fit for publication if they read the articles i have suggested and make the necessary adjustments*

RESPONSE

This is appreciated and indeed suggested articles have been consulted.

**B: SPECIFIC COMMENTS**

Reviewer #1:

1. *Line 66: ‘dairy’, not ‘diary’.*

*RESPONSE*

We thank you for this observation. We have incorporated the suggested changes (line 66).

2. *Line 107: ‘practising’ instead of ‘practicing’?*

*RESPONSE*

The comment is appreciated and we have incorporated the suggested changes (line 107).

3. *Lines 127-30, 2nd part of the sentence: change the second part of the sentence from ‘including to predict the probability of occurrence of species across space and time..’.*

*RESPONSE*

We agree with the reviewers’ observation. We have changed the suggested parts of the sentence to improve clarity and readability (*Lines 127 – 129).*

4. *Line 140: change ‘plays’ to ‘play’.*

*RESPONSE*

We thank you for this observation. We have incorporated the suggested changes (line 139).

5. *Line 141: ‘…. since blood meals are the only tsetse fly nutrition…….’.*

*RESPONSE*

We thank reviewer for this observation. Indeed, explanation given may potentially be ambiguous than we intended to communicate. We have added a language in the line 140 to 142 to indicate how precipitation is indirectly affecting tsetse fly and trypanosomes. Also line 145 to 146 has been added to improve readability and clarity.

6. *Line 209-213: ‘…… the model showed that currently 32% (19,225 km2) of the entire Maasai Steppe (≈
   60,000 km2) has suitable climate for G. m. morsitans, but this would shrink to 7.4% (4,447.34 km2) by 2050’.*

*RESPONSE*

We thank you for this observation. Indeed, the rephrasing is necessary to eliminate redundant information. Thus we have adopted reviewer’s suggestion (210 – 212) to improve the readability.

7. *Line 258-262: The three sentences appear to repeat more or less the same message……it can be
   condensed into one or two sentences.*

*RESPONSE*

We agree that, the suggested rephrasing will improve readability, we then have rephrased the sentence and eliminated unnecessary redundant information. The changes are reflected in line 257 to 260.

Reviewer #2:

1. *Page 1 ABSTRACT*

*While understanding tsetse fly distribution is essential for informing vector and disease control strategies, existing distribution maps are old and were based on coarse spatial resolution data which is not useful in understanding vector and disease dynamics necessary to design and implement fit for purpose mitigation strategies.*

*“****NOT USEFUL****” The reviewer wrote “This is an overstatement, these existing maps have guided interventions for many years, but you would rather say they are limited or rather, less accurate if your work is providing an improvement.”*

*RESPONSE*

*Reviewer might be right; to improve the readability the sentence has been rephrased into*

*“While understanding tsetse fly distribution is essential for informing vector and disease control strategies, existing distribution maps are old and were based on coarse spatial resolution data, consequently inaccurate understanding of vector and disease dynamics necessary to design and implement fit for purpose mitigation strategies This is reflected on line 20 of the raw manuscript and page 1 of the system generated PDF copy and line 20 as well.*

2. *Line 17 (article* ***the*** *between* occurrence and parasites *was highlighted)*

*RESPONSE,*

*“the” has been deleted*

3. *Line 85 “relyies”*

*RESPONSE*

We thank you for this observation. Indeed, the term as used was ambiguous; we have replaced relyies with rely as suggested (line 85)

4. *Line 95 an article a between* adopted and general has been highlighted

*RESPONSE*

We thank you for this observation. Line 95 the article has been deleted to improve readability (line 95)

5. *Line 96 an article the between climate for the tsetse fly*

*RESPONSE*

We thank you for this observation. Line 96 the article has been deleted to improve readability (line 96)

6. *Line 110* These movement increases (the s is highlighted)

*RESPONSE*

We thank you for this observation. “These movement increases” has been changed to “These movements increase (Line 110)

7. *Line 141 “Eraborate on how this temperature affects the development of tryps.”*

*RESPONSE*

We thank you for this comment. “We have added a language to show that temperature may affects differentiation and proliferation of trypanosomes and thus trypanosomes prevalence (line 140-141)

8. *Line 142 “no information is known on effects of precipitation on tsetse fly species” This is not right, read chapters 9-14 of S.G.A. Leak 1999: Tsetse Bilogy and Ecology*

*RESPONSE*

We agree with the reviewers’ observation. The sentences left the way it might lead into unnecessary confusion; thus we have refocused the literature to show specifically how rain is expected to indirectly affect tsetse fly (line 141-142; 145 - 146).

9. *Line 162 it’s is highlighted*

*RESPONSE*

We appreciate the comment. *it’s* is is now replace with it is (line 161).

10. *Line 173-175 “Mean maximum temperature of the driest month was
    omitted from analysis because of the relatively lower knowledge of biological values of dryness to tsetse fly”. SUGGESTION was “Elaborate on the values you mean here, is it survival or what? it is an unqualified statement.”*

*RESPONSE*

We thank you for this observation. Indeed, explanation given was ambiguous than we intended to communicate. We have added a language line 173 – 175 to show the existing knowledge of dryness in relation to tsetse fly and justify why it was not considered very important in the model

11. *Line 177 – 178 “Sample bias file was excluded in the model with the assumption that tsetse flies are likely to be present in large part of the study area due to widely spread of hosts”. I thought that this would take care of the micro habitats, because they are there much as tsetse may be distributed in a large area. Read Beadell et al. 2010. Also is it possible that there may be micropopoulations within the large part? Read Hyseni and Kato et al. 2012; Echodu et al 2010; Cecchi et al, 2008”*

*RESPONSE*

The comment is appreciated; however the purpose of Sample bias file is to reflect on sampling effort of the study area so that **MaxENT** can account for **sampling bias**. To ensure that *I micro habitats are considered in the model, Methodology section, subsection Species occurrence data line 114 – 119, sampling was planned and executed in light of habitat variation and thus microclimate is expected to be captured directly.*
